# Comparative Analysis of Virulence and Toxin Expression of Vancomycin-Intermediate and Vancomycin-Sensitive *Staphylococcus aureus* Strains

**DOI:** 10.3389/fmicb.2020.596942

**Published:** 2020-10-30

**Authors:** Ye Jin, Xiao Yu, Shuntian Zhang, Xiaoyang Kong, Weiwei Chen, Qixia Luo, Beiwen Zheng, Yonghong Xiao

**Affiliations:** ^1^State Key Laboratory for Diagnosis and Treatment of Infectious Diseases, National Clinical Research Center for Infectious Diseases, Collaborative Innovation Center for Diagnosis and Treatment of Infectious Diseases, The First Affiliated Hospital, Zhejiang University School of Medicine, Hangzhou, China; ^2^Department of Respiratory and Critical Care Medicine, First Hospital of Shanxi Medical University, Taiyuan, China; ^3^Department of Laboratory Medicine, College of Medicine, Zhejiang University, Hangzhou, China

**Keywords:** vancomycin-intermediate *staphylococcus aureus*, vancomycin-sensitive *staphylococcus aureus*, virulence, persistent infection, immune evasion, quorum sensing accessory gene regulator system *agr*

## Abstract

Previous studies on vancomycin-intermediate *Staphylococcus aureus* (VISA) have mainly focused on drug resistance, the evolution of differences in virulence between VISA and vancomycin-sensitive *S. aureus* (VSSA) requires further investigation. To address this issue, in this study, we compared the virulence and toxin profiles of pair groups of VISA and VSSA strains, including a series of vancomycin-resistant induced S. aureus strains—SA0534, SA0534-V8, and SA0534-V16. We established a mouse skin infection model to evaluate the invasive capacity of VISA strains, and found that although mice infected with VISA had smaller-sized abscesses than those infected with VSSA, the abscesses persisted for a longer period (up to 9 days). Infection with VISA strains was associated with a lower mortality rate in *Galleria mellonella* larvae compared to infection with VSSA strains (≥ 40% vs. ≤ 3% survival at 28 h). Additionally, VISA were more effective in colonizing the nasal passage of mice than VSSA, and *in vitro* experiments showed that while VISA strains were less virulent they showed enhanced intracellular survival compared to VSSA strains. RNA sequencing of VISA strains revealed significant differences in the expression levels of the *agr*, *hla*, *cap*, *spa*, *clfB*, and *sbi* genes and suggested that platelet activation is only weakly induced by VISA. Collectively, our findings indicate that VISA is less virulent than VSSA but has a greater capacity to colonize human hosts and evade destruction by the host innate immune system, resulting in persistent and chronic *S. aureus* infection.

## Introduction

*Staphylococcus aureus* is a major multidrug−resistant opportunistic pathogen in healthcare settings that represents a significant public health burden (Deresinski, 2005). Acquisition of the *mecA* gene encoding penicillin-binding protein 2 conferring methicillin resistance (Kobayashi et al., 2002) has led to the emergence of methicillin-resistant *S. aureus* (MRSA), which is responsible for thousands of infections annually and contributes to the growing problem of hospital-acquired (HA) infections (Oliveira et al., 2002). Glycopeptides such as vancomycin are the primary treatment option for severe infections caused by Gram-positive bacteria, including MRSA and most strains of multidrug-resistant *S. aureus* (Walsh, 1999).

Vancomycin resistance in Gram-positive bacteria was first reported in *Enterococcus faecium* in 1988 (Leclercq et al., 1988). The transfer of genetic elements containing the vancomycin resistance gene *vanA* from *E. faecalis* to *S. aureus* was discovered in 1992 (Noble et al., 1992). In 1997, the first clinical *S. aureus* strain (Mu50) with decreased susceptibility to vancomycin [minimal inhibitory concentration (MIC) = 8 μg/ml] was isolated in Japan (Hiramatsu et al., 1997; Centers for Disease Control and Prevention, 2002b), and was designated as vancomycin-intermediate *S. aureus* (VISA). At the same time, *S. aureus* strains exhibiting a heterogeneous (h)VISA phenotype (Mu3) were isolated from clinical specimens (Hiramatsu et al., 1997). The first completely resistant clinical isolate of *S. aureus* (MIC > 1,024 μg/ml) was reported in July 2002 in the United States (Centers for Disease Control and Prevention, 2002a).

Most studies on VISA/hVISA have focused on drug resistance, and the virulence of VISA/hVISA compared to vancomycin-sensitive *S. aureus* (VSSA) requires further investigation, although VISA is known to have a thicker cell wall (Cui et al., 2000; Howden et al., 2006; Katayama et al., 2009). To address this issue, the present study investigated the ability of VISA and VSSA strains to infect or colonize skin and soft tissue as well as the possible determinants of chronic infection by VISA. We compared the virulence of vancomycin-resistant *S. aureus* and VISA strains derived from Chinese HA-MRSA lineages to that of VSSA strains of the same lineage using rodent and insect infection models and a rodent colonization model along with *in vitro* assays. We also carried out a comparative RNA sequencing (RNA-seq) analysis to evaluate differences in the expression of toxin genes between VISA and VSSA.

## Materials and Methods

### Bacteria Information

Detailed information on each isolate used in the study is shown in Table 1. The clinical *S. aureus* strain SA0534 and laboratory-induced vancomycin resistance strains (SA0534-V8 and SA0534-V16) were obtained from Yuanyuan Dai at The Affiliated Provincial Hospital of Anhui Medical University (Chang et al., 2015). VS-2393 and VI-2562 strains were isolated from different patients in Xinjiang Province, while VS-1201 and VI-1130 were isolated from patients in Guangdong Province.

**TABLE 1:** The Staphylococcus aureus strains used in this study.

**Strain**	**Molecular feature**	**MIC of vancomycin (μ g/ml)**	**Source**
**Pair 1**			
VS-2393	ST239-t030, MRSA, agr4	1	Xinjiang
VI-2562	ST239-t030, MRSA, agr4	8	Xinjiang
**Pair 2**			
VS-1201	ST121-t1425, MSSA, agr4	2	Guangdong
VI-1130	ST121-t1425, MSSA, agr4	16	Guangdong
**Pair 3**			
SA0534	ST239-t030, MRSA, agr1	2	Anhui
SA0534-V8	ST239-t030, MRSA, agr1	8	Anhui
SA0534-V16	ST239-t030, MRSA, agr1	16	Anhui

*The VS- indicates that this strain belongs to VSSA. The VI- indicates that the strain belongs to VISA. The SA0534-V8 and SA0534-V16 is VISA strains while SA0534 is a VSSA strain.*

### Mouse Model of Skin Infection

The mouse skin infection model was established as previously described (Li et al., 2010) using 6-week old female BALB/C-nude mice. The mice were housed for 1 week with free access to food and water prior to infection with *S. aureus*. *S. aureus* strains were grown for 16 h, washed 3 times with sterile NaCl solution. Then 2 × 10^8^ bacterial cells were suspended in 100 μl NaCl solution. Mice were injected with the *S. aureus* suspension (100 μl) or with sterile NaCl solution as a negative control. Skin abscesses that developed in the mice were monitored daily for 9 days, and their length (L) and width (W) were measured using calipers. The abscess area was calculated as area = L × W. After all animals were euthanized, skin lesions were compared between mice infected with VISA vs. VSSA strains (*n* = 6 mice per group). The community-associated MRSA strain USA300_FPR3757 was used as a positive control for its high virulence, while the USA300△agr mutant served as the negative control.

### Survival Rates of *Galleria mellonella* Larvae Infected With VISA and VSSA

*S. aureus* strains were inoculated on blood agar medium and cultured overnight at 37°C in a thermostatic incubator. A single clone was inoculated into 5 ml of trypticase soy broth (TSB) liquid medium. After secondary activation, cells were inoculated into TSB medium and cultured at 37°C with shaking at 220 rpm until the late stage of logarithmic growth.

Activated VSSA and VISA strains were cultured to late logarithmic phase and then resuspended in NaCl solution at an optical density at 600 nm of 1.0. After weighing, large *G. mellonella* larvae were paired and grouped so that each group [VISA, VSSA, normal saline (control), and blank] was of equal quality, with an average of about 250 mg larvae per group. The concentration of bacteria in the solution injected into larvae of the VISA and VSSA groups was 1.0 × 10^7^ colony-forming units (CFU)/100 μl; the injection site was between the second and third gastropods. Repeated injections at the same site were avoided. The treated larvae were placed in a clean, sterile plastic Petri dish in a 37°C incubator. All 4 groups were grown in the same environment. The number of dead larvae was recorded for 40 h to calculate survival rate.

### Mouse Nasal Colonization Model

Phosphate-buffered saline (PBS; 20 μl) containing 1 × 10^8^ bacterial cells was introduced as a drop into the nasal cavity of mice. After 3 days, the mice were euthanized and the nose was dissected and homogenized. Total *S. aureus* count was determined by plating 200 μl diluted nose tissue suspension onto TS agar.

### Biofilm Formation

*S. aureus* strains were grown in a 96-well microplate in TSB containing 0.5% glucose at 37°C for 24 h. After 3 washes with PBS, the biofilm was fixed with 95% methanol and stained with 0.5% crystal violet dye for 20 min.

### Analysis of Hemolytic Capacity

Hemolytic capacity was assessed as previously described (Jin et al., 2018). Briefly, *S. aureus* strains were grown in TSB at 37°C to the mid-exponential growth phase (6 h). After 6 h of incubation, the cells were centrifuged at 12,800 rpm and the supernatant was collected. Rabbit red blood cells (RRBCs) were washed 3 times with PBS (pH 7.2) and then mixed with 0.1% bovine serum albumin (Sigma-Aldrich, St. Louis, MO, United States) in fresh centrifuge tubes. An aliquot of supernatant was added to RRBCs, followed by incubation at 37°C for 3 h. Triton X-100 (1%) solution was used as a positive control and RRBCs resuspended in 1 × PBS served as a negative control.

### Semiquantitative Detection of α-Toxin by Enzyme-Linked Immunosorbent Assay (ELISA)

Detection of α-toxin by ELISA was performed as previously described (Otto et al., 2013). Briefly, *S. aureus* strains were cultured overnight at 37°C. Samples were centrifuged at 12,800 rpm for 6 min. Monoclonal anti-α-hemolysin (Hla) antibody (Sigma-Aldrich) was used in sandwich-type ELISA in order to quantify the amount of α-toxin in the supernatant. Rabbit polyclonal anti-Hla antibody was used to detect the antigen–antibody complex. The linear equation y = ax + b was used to calculate the amount of α-toxin produced by each strain.

### *S. aureus* Survival in Human Whole Blood

*S. aureus* strains were grown in TSB at 37°C to the mid-exponential growth phase (6 h). After 3 washes in sterile NaCl solution, cells were resuspended in sterile PBS (pH 7.2) at 10^7^ CFU/ml. A 100–μl aliquot of bacterial solution was added to 10 ml heparinized blood and incubated at 37°C. To determine the survival rate of bacteria in whole blood, 100 μl of heparinized blood at different time points (0, 0.5, 1, 1.5, 2, and 3 h) was plated on TS agar and colonies were counted.

### Analysis of Cell Viability With the Lactate Dehydrogenase (LDH) Assay

Human neutrophils were obtained from the venous blood of healthy volunteers and mixed with heparin using a standard method (Kobayashi et al., 2002) in accordance with a protocol approved by the ethics committee of The First Affiliated Hospital of Zhejiang University, College of Medicine. Each volunteer provided informed consent before donating blood. Strains were grown for 6 h, and neutrophils were added at a 10:1 ratio (i.e., multiplicity of infection of 10), followed by incubation at 37°C for 3 h. PBS with 0.1% Triton-X-100 (100 μl) was used to as a positive control as it induces 100% lysis. Cell viability was determined using Cytotoxicity Detection KitPLUS (LDH) (Roche, Welwyn Garden City, United Kingdom) according to the manufacturer’s instructions. The absorbance of each well was measured to calculate the cytotoxicity to each strain after subtracting the background absorbance value according to the following formula:

$$Cytotoxicity\left(\%\right)=\left(\frac{\left[E\,x\,p\,e\,r\,i\,m\,e\,n\,t\,a\,l\,v\,a\,l\,u\,e-L\,o\,w\,c\,o\,n\,t\,r\,o\,l\right]}{\left[H\,i\,g\,h\,c\,o\,n\,t\,r\,o\,l-L\,o\,w\,c\,o\,n\,t\,r\,o\,l\right]}\right)\times 100$$

### RNA Isolation and Sequencing

*S. aureus* strains were grown at 37°C with shaking at 220 rpm. After 9 h, the cultures were centrifuged at 12,800 × *g* for 10 min at 4°C. The supernatant was transferred to a vial containing 1.5 ml of 0.1 mm zirconia silica beads. Total RNA was released by ultrasonic disruption using a Mini-Bead Beater (BioSpec Products, Bartlesville, OK, United States). The RNeasy Plus Mini Kit (Qiagen, Hilden, Germany) was used according to the manufacturer’s instructions to purify RNA from each strain. RNase-free DNase I (Takara Bio, Otsu, and Japan) was used to eliminate contaminating genomic DNA and rRNA was removed prior to sequencing. RNA-seq of 3 biological replicates of SA0534 and SA0534-V16 was performed by Novogene Co. (Beijing, China) after samples were tested for purity. Differential gene expression was determined according to the following criteria: | log2 (fold change)| > 1, *p* < 0.05, and *q* < 0.05.

### Statistical Analysis

ELISA and hemolytic test results were analyzed with the chi-squared test. Survival rates of *G. mellonella* larvae infected with VSSA and VISA were determined by Kaplan–Meier analysis. Percent survival in human blood and cytotoxicity assay results were analyzed with the paired-samples *t*-test. Results of the quantitative analysis of nasal colonization and biofilm formation were evaluated with the *t*- and *q*-tests. Two-way factorial analysis of variance was used to assess differences in skin lesions caused by VISA and VSSA strains in mice. Statistical analyses were performed using Prism v7.0 software (GraphPad, San Diego, CA, and United States), and data are reported as mean ± standard error of the mean.

## Results

### *S*. *aureus* Strain Information

All clinical *S. aureus* isolates were isolated in the operation room and hematology and urinary surgery wards of the hospital. VS-1201 and VI-1130 were identified as ST121-MSSA, whereas VS-2393 and VI-2562 were identified as ST239-MRSA-III-t030, which is the predominant HA–MRSA strain in China. A series of VISA strains were derived from SA0534. The isolates with MIC of vancomycin at 8 and 16 mg/l were named SA0534-V8 and SA0534-V16, respectively. We also identified SA0534 as a t030-carrying ST239 SCC*mec* III isolate.

### Mouse Skin Infection Model

**FIGURE 1:**
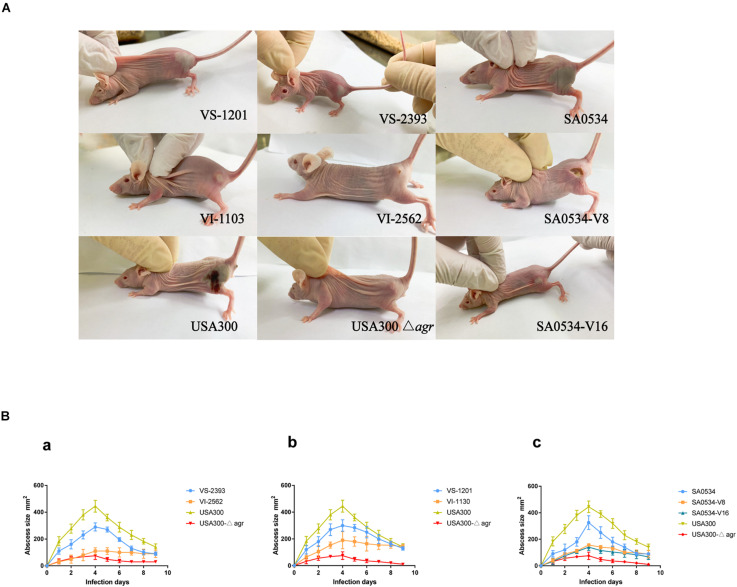
Results of mouse skin infection model experiments. **(A)** Is the photo of the comparison of abscess area of BALB/C-nude mice infected with VSSA and VISA strains. **(B)** Is line chart of the abscess area. Each strain injected six mice. The VS- indicates that this strain belongs to VSSA. The VI- indicates that the strain belongs to VISA. The SA0534-V8 and SA0534-V16 is VISA strains while SA0534 is a VSSA strain. Two-way analysis of variance was used to compare data for multiple groups.

We compared the infectivity of VISA and VSSA using a mouse abscess model. Skin lesions in mice caused by VISA and VSSA infection are shown in Figure 1A. In the pair 1 group, mice infected with VS-2393 had mid-sized abscesses 0.8–1.6 cm in diameter, whereas VI-2562 caused almost no abscess formation (Figure 1Ba). In the pair 2 group, mice infected with the VS-1201 strain had abscesses with a diameter 150 mm larger than those produced by VI-1130. On day 4 after injection, when the maximum range of skin lesion area was observed, the average abscess area in mice injected with the SA0534-V8 and SA0534-V16 strains was significantly smaller than that of mice infected with the SA0534 strain (Figure 1Bc). Moreover, there were no statistically significant differences between mice infected with the SA0534-V8 and SA0534-V16 strains. On day 9 after infection, the size of abscesses in mice infected with VSSA was significantly diminished. In contrast, abscesses were still observed in the VISA groups and there was no visible reduction in abscess area.

### G. *mellonella* Infection Model

**FIGURE 2:**
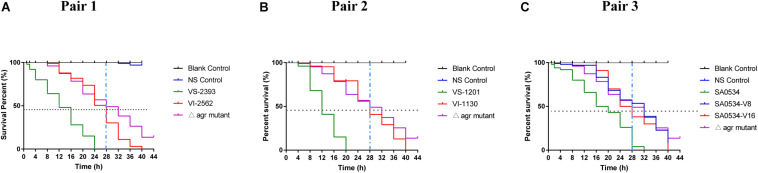
Comparison of survival rates of VSSA and VISA infected with *Galleria mellonella* larvae. *G*. *mellonella* larvae were inoculated with 10 μl each pair at doses ranging from 1 × 10^4^ and incubated at 37°C; the viability was assessed over 40 h. The VS- indicates that this strain belongs to VSSA. The VI- indicates that the strain belongs to VISA. The SA0534-V8 and SA0534-V16 is VISA strains while SA0534 is a VSSA strain. **(A)** Is the survival rates of VS-2393 and VI-2562 infected with *G. mellonella* larvae. **(B)** Is the survival rates of VS-1201 and VI-1130 infected with *G. mellonella* larvae. **(C)** Is the survival rates of SA0534, SA0534-V8, and SA0534-V16 infected with *G. mellonella* larvae.

The *G. mellonella* infection model was used in this study as a relatively rapid and potentially high-throughput *in vivo* assay compared to mammalian models that has been applied to investigations of *S. aureus* virulence (Peleg et al., 2009; Ramarao et al., 2012). Based on the results obtained with the mouse skin infection model, we compared survival rates of *G. mellonella* infected with VISA and VSSA strains to evaluate differences in pathogenicity. In each pair group, the percentage of surviving larvae was much higher following infection with VISA as compared to VSSA strains (*p* < 0.01 for each pair group) (Figure 2). The SA0534-V8 and SA0534-V16 strains had comparable effects on the survival of *G. mellonella*, which was similar to that of the negative (*agr* mutant) control (Figure 2C). The SA0534 strain was more virulent than the SA0534-V8 and SA0534-V16 strains (*G. mellonella* survival ≤ 52% at 12 h and ≤ 3% at 28 h). Meanwhile, the survival rates of VSSA strains VS-1130 and VI-2562 were higher than those of VISA strains.

### VISA Has a Higher Adhesive Capacity Than VSSA

**FIGURE 3:**
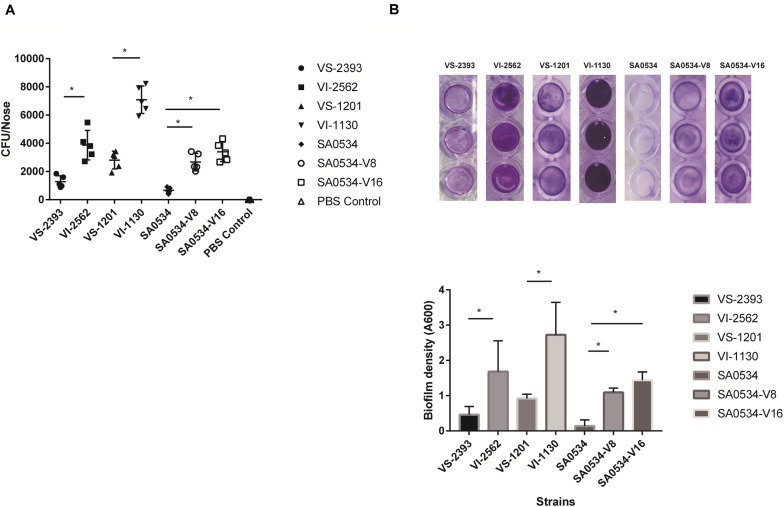
The comparison of nasal colonization and biofilm formation ability of VISA and VSSA strains. Values are means ± SD (three repeated different experiments). *P < 0.05. The VS- indicates that this strain belongs to VSSA. The VI- indicates that the strain belongs to VISA. The SA0534-V8 and SA0534-V16 is VISA strains while SA0534 is a VSSA strain. **(A)** Is the nasal colonization ability of VISA strains in mice (*n* = 5) compared with that of VISA strains. **(B)** Is the biofilm formation ability of VISA and VSSA strains.

*S. aureus* is an opportunistic pathogen that mainly colonizes healthy human nasal mucosa. The capacity of *S. aureus* for adhesion and colonization plays an important role in chronic infection (Rollin et al., 2017; Tan et al., 2019). We therefore compared nasal colonization by VISA and VSSA strains in BALB/c mice. All VISA strains showed a higher capacity for nasal colonization (Figure 3A) and biofilm formation (Figure 3B) than VSSA strains (both *p* < 0.05), reflecting a greater capacity for adhesion.

### VISA Has Lower α-Toxin Activity Than VSSA

**FIGURE 4:**
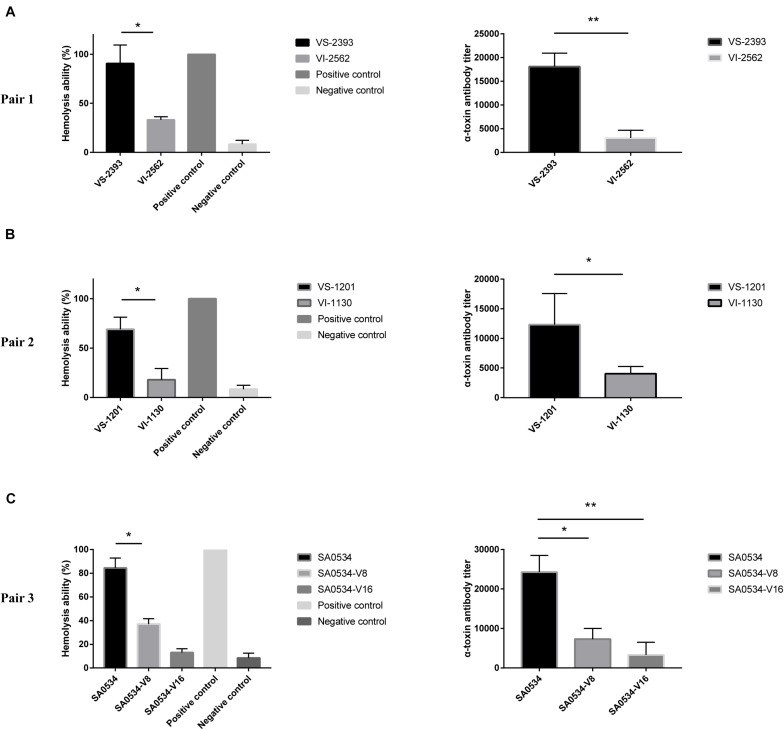
α-toxin activity and production between VSSA and VISA strains. Spectrophotometer was used to calculated the absorbance (A600 nm) of each sample. The value of complete hemolysis group (positive control) was set as 100%. ELISA was used to semi-quantify the concentration of α-toxin. Supernatant without antibody was used as a negative control. Less than twice the value of the negative control was defined as the cut-off value. Values are means ± SD (three repeated different experiments). *P < 0.05, **P < 0.01. The VS- indicates that this strain belongs to VSSA. The VI- indicates that the strain belongs to VISA. The SA0534-V8 and SA0534-V16 is VISA strains while SA0534 is a VSSA strain. **(A)** Is the α-toxin activity and production between VS-2393 and VI-2562. **(B)** Is the α-toxin activity and production between VS-1201 and VI-1130. **(C)** Is the α-toxin activity and production among SA0534, SA0534-V8, and SA0534-V16.

Previous studies have shown that VISA has lower virulence than VSSA (Majcherczyk et al., 2008; Cameron et al., 2017). We compared the α-toxin activities of VISA and VSSA strains and found that in each pair group, α-toxin activity was lower in VISA than in VSSA (*p* < 0.05) (Figure 4). Furthermore, 74.26% (VS-1201) and 88.6% (VS-2393) of RRBCs were lysed by α-toxin, with 8.85- to 3.60-fold higher antibody titers than for VSSA strains (Figures 4A,B). The α-toxin activity of VISA strains (VI-1130 and VI-2562) led to significant reductions in the number of RRBCs (3.53 and 2.84 fold). As variability in the genetic background of VS-1201-SA1130 and VS-2393-VI-2562 could potentially contribute to the observed differences in virulence between VISA and VSSA, we further investigated the hemolytic capacity of a series of strains with induced vancomycin resistance (SA0534-V8 and SA0534-V16) in RRBCs. The α-toxin activities of strains SA0534-V8 (11.52%) and SA0534-V16 (18.46%) were lower than that of isolate SA0534 (79.26%), with a 6.53-fold lower antibody titer (Figure 4C).

### VISA Is Less Cytotoxic to Human Neutrophils Than VSSA

**FIGURE 5:**
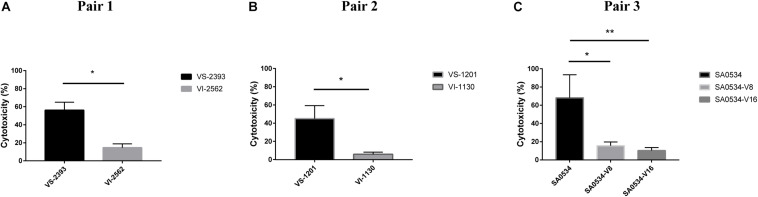
Intracellular cytotoxicity of VSSA and VISA strains in human neutrophils. We performed the cytotoxicity assay, following controls. (1) Exp. value: the absorbance value of each experimental sample. (2) Low control: the absorbance value of culture medium background. (3) High control: the absorbance value of the positive control (maximum LDH release). Values are means ± SD (three repeated different experiments). *P < 0.05, **P < 0.01. The VS- indicates that this strain belongs to VSSA. The VI- indicates that the strain belongs to VISA. The SA0534-V8 and SA0534-V16 is VISA strains while SA0534 is a VSSA strain. **(A)** Is the comparison of cytotoxicity between VS-2393 and VI-2562. **(B)** Is the comparison of cytotoxicity between VS-1201 and VI-1130. **(C)** Is the comparison of cytotoxicity among SA0534, SA0534-V8, and SA0534-V16.

Human neutrophils are part of the innate immune system, which fights infection by pathogens. We evaluated differences in the pathogenicity of *S. aureus* by comparing the cytotoxicity of VISA and VSSA *in vitro* based on the level of reduced LDH released by damaged neutrophils after phagocytosis by *S. aureus* strains. The degree of cellular damage caused by VSSA (VS-1201 and VS-2393) was 3.86-fold higher than that caused by VISA strains (VS-1130 and VI-2562) (*p* < 0.05) (Figure 5A). We also evaluated the viability of the induced vancomycin resistance strain SA0534 and found that it caused more cellular damage than SA0534-V8 and SA0534-V16 (*p* < 0.05) (Figure 5B). These results indicate that VSSA is more toxic to human neutrophils than VISA.

### VISA Has Higher Viability in Human Blood Than VSSA

**FIGURE 6:**
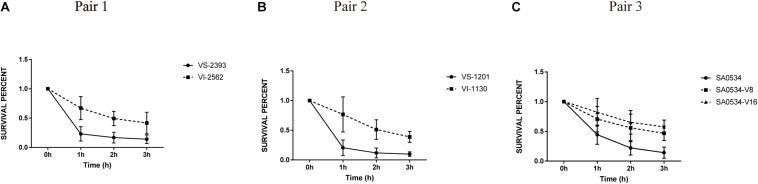
The survival percentage in human blood between VISA strains and VSSA strains. The number of CFU was detected at the point at 0–3 h to calculate the rates of survival for three independent VSSA and VISA pairs exposed to human blood. Each test was repeated independently. Values are means ± SD (three repeated different experiments). The VS- indicates that this strain belongs to VSSA. The VI- indicates that the strain belongs to VISA. The SA0534-V8 and SA0534-V16 is VISA strains while SA0534 is a VSSA strain. **(A)** Is the comparison of survival percentage in human blood between VS-2393 and VI-2562. **(B)** Is the comparison of survival percentage in human blood between VS-1201 and VI-1130. **(C)** Is the comparison of survival percentage in human blood among SA0534, SA0534-V8, and SA0534-V16.

To further assess the virulence and pathogenicity of VISA, we compared the relative viabilities of VISA and VSSA strains in human blood. The percent survival of the VSSA strains VS-1201 and VS-2393 was 2.34- and 3.18-fold lower than that of the VISA strains VI-1130 and VI-2562, respectively, when cultured for 1 h (Figures 6A,B). After 3 h of culture, survival rates were lower for VSSA groups (14.28 and 9.85%) than for VSSA groups (41.73 and 33.77%); and the survival of isolates SA0534-V8 (46.82%) and SA0534-V16 (57.53%) was higher than that of isolate SA0534 (14.17%) (Figure 6C). Notably, the survival of VISA strains was inversely related to vancomycin MIC (*p* < 0.05). Thus, compared to VSSA, VISA has lower invasive capacity and cytotoxicity toward human neutrophils but shows enhanced ability to evade human immune surveillance. These findings are significant because clinical isolates of VISA often cause chronic and persistent infections.

### Transcriptome Profiling of VISA and VSSA

We identified 512 differentially expressed genes (DEGs) between the SA0534-V16 and SA0534 strains; of these, 261 were upregulated and 251 were downregulated in VISA as compared to VSSA strains (Supplementary Tables S1, S2). We selected 30 DEGs for validation by real-time quantitative PCR and found that their expression levels were consistent with the transcriptome profiles.

The gene *lrgAB* encoding a murein hydrolase regulator essential for cell lysis was downregulated 7.11 and 4.89-fold in VISA strains compared with VSSA, respectively; *atlA*, an autolysin protein-encoding gene was downregulated 8.13-fold relative to its level in the VSSA strain. AtlA induces platelet aggregation in whole blood (Binsker et al., 2018). Expression of *hla* encoding an *S. aureus* virulence factor was much lower in VISA as compared to VSSA. The *agr* genes (agrABCD) play an important role in regulating *S. aureus* virulence factors; we found here that *agrA*, *agrB*, and *agrC* were downregulated 6.40, 2.28, and 5.98-fold, respectively, in SA0534 relative to SA0534-V16.

The level of *sbi* encoding the immunoglobulin G-binding protein was decreased in the SA0534 strain compared to the SA0534-V16 strain (Table 2). Meanwhile, expression of capsular polysaccharide-associated genes (*cap8B*, *cap8C*, *cap8D*, *cap8E*, *cap8F*, and *cap8M*) was elevated in the VISA strain. These genes are part of a global transcriptional response that contributes to evasion of innate immune surveillance (Voyich et al., 2005).

**TABLE 2:** Genes differentially expressed between the VISA and VSSA strains.

**Gene_ID**	**log_2_ fold change**	**RT-qPCR**	***P*-value**	***q*-value**	**Description**
**Autolysin-associated genes**					
*SAOUHSC_01524*	–6.4203	–2.10	2.86E-11	5.14E-11	Holin-like protein
*SAOUHSC_02019*	–8.813	–3.12	4.91E-42	2.57E-41	Autolysin
*SAOUHSC_00232*	–2.8307	–1.39	4.62E-33	2.05E-32	Murein hydrolase regulator LrgA
*SAOUHSC_00233*	–2.2949	–5.92	3.18E-56	2.01E-55	Anti-holin-like protein LrgB
**Cell wall-associated genes**					
SAOUHSC_00069	–4.6639	–7.32	2.34E-46	1.30E-45	Protein A Spa
SAOUHSC_00229	–2.172	–4.37	0	0	Cell wall biosynthesis protein ScdA
SAOUHSC_01110	2.6238	1.04	1.01E-23	3.58E-23	Fibrinogen-binding protein-like protein
SAOUHSC_02802	4.2929	ND	7.78E-06	1.18E-05	Fibronectin binding protein B
SAOUHSC_01142	–2.4371	–1.34	1.58E-93	1.47E-92	Cell division protein MraZ
SAOUHSC_02963	–2.5384	–2.98	1.45E-17	4.14E-17	Clumping factor B ClfB
SAOUHSC_00544	2.9992	1.50	4.01E-29	1.44E-28	Fibrinogen-binding protein SdrC
SAOUHSC_00545	2.1248	3.96	6.55E-10	1.13E-09	Fibrinogen-binding protein SdrD
SAOUHSC_00114	8.0625	5.83	0	0	Capsular polysaccharide biosynthesis protein Cap5A
SAOUHSC_00115	7.5579	4.93	0	0	Capsular polysaccharide biosynthesis protein Cap5B
SAOUHSC_00116	8.2873	2.34	0	0	Capsular polysaccharide biosynthesis protein Cap8C
SAOUHSC_00117	9.0369	ND	0	0	Capsular polysaccharide biosynthesis protein Cap5D
SAOUHSC_00118	8.9985	2.40	2.08E-88	1.89E-87	Capsular polysaccharide biosynthesis protein Cap5E
SAOUHSC_00119	7.6467	4.69	0	0	Capsular polysaccharide biosynthesis protein Cap8F
SAOUHSC_00126	6.0723	7.23	7.07E-33	2.77E-32	Capsular polysaccharide biosynthesis protein Cap8M
SAOUHSC_00176	–6.6897	–2.98	0	0	Extracellular solute-binding protein
**Virulence-associated genes**					
SAOUHSC_01121	–4.1632	–6.23	0	0	Alpha-hemolysin
SAOUHSC_01942	–4.4034	–2.01	0.00013251	0.00016629	Serine protease SplA
SAOUHSC_01941	–3.2598	–3.29	0.0003595	0.00042238	Serine protease SplB
SAOUHSC_01939	–3.7964	–1.23	2.73E-08	4.78E-08	Serine protease SplC
SAOUHSC_00300	–2.4787	–1.03	2.09E-39	1.04E-38	Lipase
SAOUHSC_02706	–2.0555	–6.25	2.26E-10	4.59E-10	Immunoglobulin G-binding protein Sbi
SAOUHSC_00177	–6.0315	ND	0	0	Maltose ABC transporter permease
**Hypothetical protein**					
SAOUHSC_00179	–6.4487	ND	0	0	Hypothetical protein
SAOUHSC_00180	–6.1303	–0.2.98	0	0	Hypothetical protein
SAOUHSC_00208	–7.054	ND	1.13E-36	4.73E-36	Hypothetical protein
SAOUHSC_00049	–8.0233	–4.92	4.32E-30	1.57E-29	Hypothetical protein
**Transcriptional regulators**					
SAOUHSC_02265	–6.3987	–3.25	1.39E-56	1.35E-55	Accessory gene regulator protein A
SAOUHSC_02261	–2.2777	–4.24	2.80E-35	1.63E-34	Accessory gene regulator protein B
SAOUHSC_02264	–5.9723	–1.53	0	0	Accessory gene regulator protein C
SAOUHSC_00469	1.3894	ND	0	0	Regulatory protein SpoVG
SAOUHSC_02315	2.5381	ND	1.88E-05	2.33E-05	DNA-binding response regulator
SAOUHSC_02862	1.087	3.72	0	0	ATP-dependent Clp protease ATP-binding subunit ClpC

*ND, not done.*

Expression of Staphylococcal protein A (SpA) and clumping factor B (ClfB) was lower in the VISA strain than in the VSSA strain. Sbi promotes evasion of human neutrophil-mediated phagocytosis (Foster, 2005), while *spA* can bind to the Fc region of IgG to inhibit opsonophagocytosis, thus preventing activation of the classical complement pathway of the immune system and recognition by the neutrophil Fc receptor (Foster, 2005, 2009). ClfB is a major fibronectin-binding surface protein (Josefsson et al., 1998; Zhu et al., 2019) A previous study showed that the *S. aureus clfB* mutants were significantly attenuated in mouse sepsis models, arthritis models (McDevitt et al., 1997; O’Brien et al., 2002) and in a rat endocarditis model (Que et al., 2001). The Spl operon is unique to *S. aureus*, and comprises genes encoding 6 serine proteases on the νSaβ pathogenicity island. The spl operon has been implicated in localized lung damage, and *spl* mutants show altered expression of secreted and surface-associated proteins (Paharik et al., 2016). In this study, *splA*, *splB*, and *splC* levels were decreased 21.11, 9.51, and 13.83-fold, respectively. These data suggest that the production of extracellular secreted proteins encoded by *hla* and splABC and immune evasion proteins encoded by *sbi*, *spa*, and *clfB* is reduced in VISA compared to VSSA, while the opposite is true for *fnbB* and sdrCDE. *fnbB* is an important virulence gene that has been shown to contribute to *S. aureus* persistence on cardiac valves in experimental endocarditis (Que et al., 2005). Additionally, sdrCDE can induce *staphylococcal* biofilm formation and contributed to the ability of *S. aureus* to adhere to squamous cells (Corrigan et al., 2009; Barbu et al., 2014; Askarian et al., 2016).

## Discussion

Vancomycin is the first-line antibiotic for the treatment of MRSA infection. However, the excessive use of vancomycin has led to the emergence of VISA and hVISA strains worldwide. hVISA/VISA strains have a thicker cell wall compared to VSSA strains (Moreira et al., 1997; Boyle-Vavra et al., 2001), as well as lower autolytic activity (Dai et al., 2017) and altered cell wall-associated protein profile, growth rate, and Agr system function (Cui et al., 2003; Dai et al., 2017). Several studies have reported differences in pathogenicity between VISA and VSSA, with the former showing less hemolytic activity and producing less α-toxin, thus showing less cytotoxicity and virulence in an animal model (Cameron et al., 2017). VISA is associated with persistent and chronic infections such as bacteremia rather than acute clinical instability or lethal sepsis (Hegde et al., 2010). This suggests that although VISA is less virulent than VSSA, it can cause persistent infection.

The *G. mellonella* infection model has been widely used to compare the virulence of VISA and VSSA (Peleg et al., 2009). We used this model to determine whether VISA strains cause more persistent infection than VSSA strains. The results revealed differences in the pathogenicity of VISA and VSSA, with the latter being more lethal to *G. mellonella* larvae. In a mouse model of skin and soft tissue infection with *S. aureus*, abscess size was smaller on day 4 in mice infected with VISA as compared to VSSA. Notably, abscess formation persisted in VISA-infected mice up to day 9. These findings demonstrate that in acute skin and soft tissue infections, the invasion capacity of VISA is much lower than that of VSSA; and in chronic and persistent infections, VISA elicits innate immune system activation to a lesser extent than VSSA. This may be explained by lower or altered *agr* expression in VISA, as inactivating mutations in the *agr* gene of *S. aureus* have been linked to poorer outcomes in infected patients (Smyth et al., 2012; Suligoy et al., 2018).

Biofilm formation plays an important role in persistent infection, antibiotic resistance, and immune evasion by *S. aureus* (Archer et al., 2011). A strong ability to colonize can promote progression to chronic infection. However, findings regarding VISA biofilms are controversial. Clinical VISA strains were found to have diminished capacity for biofilm formation compared to the parent VSSA strains (Sakoulas et al., 2002), but another study showed that VISA formed thicker biofilms than VSSA (Howden et al., 2006). We found here that VISA strains had greater capacity for biofilm formation than VSSA strains, and *in vivo* experiments in mice demonstrated that nasal colonization by the former was most likely related to their enhanced biofilms. This was supported by the upregulation of genes (sdrCDE, *fnbB*, and some cell-wall related genes) associated with adhesion and biofilm formation in VISA. Fibronectin binding protein B (FnbBp) is a large multidomain protein encoded by *fnbB* (Xiong et al., 2015), while SdrCDE protect bacteria from host immune mechanisms and the effects of antibiotics (Barbu et al., 2014; Zhang et al., 2017; Pi et al., 2020).

α-Toxin is a major virulence factor that plays an important role in skin and soft tissue infections (Kennedy et al., 2010; Kobayashi et al., 2011). Previous studies have reported that Hla is involved in skin infection (Kennedy et al., 2010; Chen et al., 2015; Li et al., 2016). To determine whether VISA and VSSA have comparable ability to cause skin and soft tissue infection, we evaluated α-toxin production and found that it was lower in VISA isolates, which is consistent with our findings using the mouse skin and soft tissue infection model and with the decreased expression of *hla* and *agr*, as well as the earlier observation that Hla protein expression is lower in VISA than in VSSA strains (Cameron et al., 2017).

Compared to VSSA, we found that VISA strains were less invasive and cytotoxic to human neutrophils, in accordance with their lower virulence. However, in human blood, VISA strains had higher survival rates than VSSA strains. VISA is known to have a thicker cell wall (Cui et al., 2003; Howden et al., 2006; Katayama et al., 2009). Therefore, this is likely attributable to the fact that the thicker cell wall of VISA strains comprises more peptidoglycans that confer resistance to phagocytosis by human polymorphonuclear (PMN) leukocytes, thereby increasing the intracellular survival of VISA.

We carried out an RNA-seq analysis to investigate the genetic basis for the lower pathogenicity of VISA and found that the expression of *hla* and *agrA* was decreased in VISA relative to VSSA. AgrA directly regulates the expression of RNAII and RNAIII from the P2 and P3 promoters, and upregulation of RNAIII is associated with increased production of virulence factors, including α-toxin (Hla), serine proteases (SplA-F), and lipase (Geh) (Dunman et al., 2001; Cheung et al., 2011). Additionally, changes in *agr* transcription have been reported in hVISA/VISA strains (McGuinness et al., 2017). Thus, the decrease in the α-toxin activity is likely the result of *hla* gene regulation by Agr, which can also explain the fewer skin lesions associated with VISA infection.

The dysregulation of *agr* in *S. aureus* in the progression from acute to persistent infection has been reported (Smyth et al., 2012; Painter et al., 2014; Suligoy et al., 2018). In a rat osteomyelitis model, the persistence of *S. aureus* in bone was associated with decreased expression of *agr*, although *agr* mutation reduced the capacity of *S. aureus* to induce acute inflammation (Suligoy et al., 2018). Moreover, most clinical cases of *S. aureus* bacteremia are caused by *agr*-dysfunctional *S. aureus*. In this study, the downregulation of *agr* in VISA strains may be a trade-off for reduced virulence under the stress of antibiotic treatment.

Transcriptional profiling also revealed lower expression of *spa*, *sbi*, *hla*, *clfB*, and *atlA* and higher levels of capsular polysaccharide genes in VISA as compared to VSSA. Protein A binds to the Fc region of IgG to trigger platelet aggregation (Hawiger et al., 1972), while AtlA (Binsker et al., 2018) and Sbi (Haupt et al., 2008) are critical for platelet activation, which leads to the release of platelet microbiocidal proteins (Yeaman and Bayer, 1999). Thus, the decreased expression of *spa*, *atlA*, and *sbi* in VISA may result in reduced platelet activation; additionally, the lower levels of *spa* and *sbi* may modulate immune system response and promote persistent infection.

Overproduction of staphylococcal type 8 capsular polysaccharide was shown to protect bacteria against opsonophagocytic damage by human PMN leukocytes *in vitro* and cause persistent and chronic infection in mice (Luong et al., 2002). This suggests that transcriptional changes in capsular polysaccharide genes are an adaptation that allows VISA to persist in the human body while evading immune surveillance mechanisms. Increased expression capsular polysaccharide genes can prevent ClfB-mediated binding of staphylococci to fibrinogen and platelets (Risley et al., 2007). Notably, in our study *clfB* expression was much lower in VISA strains than in VSSA strains.

A limitation of our study is that we did not address the precise contribution of individual genetic mutations to the observed difference in virulence between VISA and VSSA, although mutations in *agr*, *sbi*, *spa*, and *cap* among other genes have been reported in VISA strains (Howden et al., 2006., 2011; Gardete et al., 2012; Park et al., 2012; Passalacqua et al., 2012; Hattangady et al., 2015). However, we discussed the decreased expression of these genes in VISA in the context of clinical outcomes (e.g., reduced platelet activation and chronic infections). The full complement of pathways involved in the virulence of VISA and VSSA strains remains to be elucidated in future studies.

In conclusion, we found that the virulence of VISA strains is much lower than that of VSSA strains in acute infection, consistent with the previous studies. However, in chronic and persistent infections, VISA elicits a weaker innate immune response compared to VSSA; this is associated with changes in the expression of capsule polysaccharide genes as well as *spA*, *clfB*, and *Sbi*, as well as Agr dysfunction. VISA strains also formed thicker biofilms than VSSA strains, which can enhance colonization. Thus, VISA is less capable of causing acute infections than VSSA; however, once in the bloodstream, VISA can cause chronic infection through strong adhesion and immune evasion despite its relatively low pathogenicity. These results provide insight into the mechanisms of virulence in drug-resistant *S. aureus* and can potentially explain treatment failure in cases of HA-VISA infection (chronic infection) without an associated increase in mortality (low virulence).

## Data Availability Statement

The raw data supporting the conclusions of this article will be made available by the authors, without undue reservation, to any qualified researcher.

## Ethics Statement

The studies involving human participants were reviewed and approved by The First Affiliated Hospital of Zhejiang University, College of Medicine. The patients/participants provided their written informed consent to participate in this study. The animal study was reviewed and approved by The First Affiliated Hospital of Zhejiang University, College of Medicine.

## Author Contributions

YJ, XY, and WC designed of the work and analyzed and interpreted of data for the work. BZ and YJ drafted the work and revised it critically for important intellectual content. XY provided approval for publication of the content. XK and SZ participated in the experimental design and data analysis. XY agreed to be accountable for all aspects of the work in ensuring that questions related to the accuracy or integrity of any part of the work are appropriately investigated and resolved. All authors read and approved the final manuscript.

## Conflict of Interest

The authors declare that the research was conducted in the absence of any commercial or financial relationships that could be construed as a potential conflict of interest.
