# Supplementary material for: Comparative Analysis of Virulence and Toxin Expression of Vancomycin-Intermediate and Vancomycin-Sensitive Staphylococcus aureus Strains
Source: Front Microbiol. 2020 Oct 30;11:596942. doi: 10.3389/fmicb.2020.596942 (PMC7661696; doi:10.3389/fmicb.2020.596942)
Supplement: Supplementary file 1 [file Data_Sheet_1.docx]

**Table S1. Genes differentially expressed (the reduced expression) between the VISA and VSSA strains.**

| **Gene id** | **log2.Fold_change.** | **pvalue** | **qvalue** | **Description** |
| --- | --- | --- | --- | --- |
| SAOUHSC_00019 | -2.4441 | 2.72E-20 | 7.60E-20 | adenylosuccinate synthetase |
| SAOUHSC_00049 | -8.0233 | 4.32E-30 | 1.57E-29 | hypothetical protein |
| SAOUHSC_00050 | -1.3063 | 0.000833 | 0.000838 | hypothetical protein |
| SAOUHSC_00056 | -1.0306 | 8.00E-08 | 1.20E-07 | hypothetical protein |
| SAOUHSC_00069 | -4.6639 | 2.34E-46 | 1.30E-45 | protein A |
| SAOUHSC_00070 | -2.3231 | 8.64E-31 | 3.23E-30 | accessory regulator-like protein |
| SAOUHSC_00085 | -1.3468 | 0.001014 | 0.001008 | hypothetical protein |
| SAOUHSC_00094 | -2.6673 | 0 | 0 | hypothetical protein |
| SAOUHSC_00175 | -8.4879 | 9.97E-68 | 6.75E-67 | multiple sugar-binding transport ATP-binding protein |
| SAOUHSC_00176 | -6.6897 | 9.52E-135 | 1.25E-133 | extracellular solute-binding protein |
| SAOUHSC_00177 | -6.0315 | 1.0907E-307 | 3.66E-306 | maltose ABC transporter permease |
| SAOUHSC_00178 | -6.1701 | 3.93E-136 | 5.32E-135 | maltose ABC transporter permease |
| SAOUHSC_00179 | -6.2003 | 1.08E-287 | 3.37E-286 | hypothetical protein |
| SAOUHSC_00180 | -6.4487 | 0 | 0 | hypothetical protein |
| SAOUHSC_00181 | -6.1303 | 0 | 0 | hypothetical protein |
| SAOUHSC_00183 | -1.5968 | 2.96E-58 | 1.81E-57 | sugar phosphate antiporter |
| SAOUHSC_00187 | -1.6742 | 0 | 0 | formate acetyltransferase |
| SAOUHSC_00188 | -1.482 | 0 | 0 | pyruvate formate-lyase 1 activating enzyme |
| SAOUHSC_00195 | -1.5636 | 0.001748 | 0.001675 | acetyl-CoA acetyltransferase |
| SAOUHSC_00196 | -2.1198 | 1.71E-05 | 2.14E-05 | hypothetical protein |
| SAOUHSC_00197 | -3.1598 | 4.40E-06 | 5.70E-06 | hypothetical protein |
| SAOUHSC_00198 | -2.1287 | 9.94E-07 | 1.36E-06 | hypothetical protein |
| SAOUHSC_00199 | -1.5673 | 5.35E-30 | 1.94E-29 | hypothetical protein |
| SAOUHSC_00208 | -7.054 | 1.13E-36 | 4.73E-36 | hypothetical protein |
| SAOUHSC_00211 | -2.1988 | 2.92E-42 | 1.39E-41 | hypothetical protein |
| SAOUHSC_00215 | -2.1417 | 0.002855 | 0.002622 | PTS system transporter |
| SAOUHSC_00216 | -2.2765 | 1.51E-08 | 2.42E-08 | PTS system transporter |
| SAOUHSC_00217 | -2.8417 | 3.79E-09 | 6.33E-09 | sorbitol dehydrogenase |
| SAOUHSC_00219 | -2.7714 | 6.33E-14 | 1.32E-13 | hypothetical protein |
| SAOUHSC_00229 | -2.172 | 0 | 0 | cell wall biosynthesis protein ScdA |
| SAOUHSC_00234 | -2.9037 | 8.39E-15 | 1.86E-14 | hypothetical protein |
| SAOUHSC_00239 | -1.8725 | 2.58E-13 | 5.21E-13 | ribokinase |
| SAOUHSC_00241 | -2.1233 | 3.15E-16 | 7.40E-16 | hypothetical protein |
| SAOUHSC_00250 | -2.2832 | 0.000123 | 0.000138 | hypothetical protein |
| SAOUHSC_00251 | -1.7327 | 0.001233 | 0.001213 | hypothetical protein |
| SAOUHSC_00253 | -1.9369 | 1.69E-48 | 9.12E-48 | hypothetical protein |
| SAOUHSC_00257 | -1.5545 | 0 | 0 | hypothetical protein |
| SAOUHSC_00272 | -5.6274 | 1.03E-07 | 1.54E-07 | hypothetical protein |
| SAOUHSC_00284 | -5.0782 | 9.00E-141 | 1.30E-139 | 5'-nucleotidase |
| SAOUHSC_00290 | -1.3035 | 8.17E-06 | 1.04E-05 | hypothetical protein |
| SAOUHSC_00293 | -1.2272 | 0.001093 | 0.001082 | hypothetical protein |
| SAOUHSC_00300 | -2.4787 | 2.09E-39 | 1.04E-38 | lipase |
| SAOUHSC_00317 | -1.0147 | 1.73E-20 | 4.87E-20 | glycerol-3-phosphate transporter |
| SAOUHSC_00324 | -1.2429 | 7.65E-05 | 8.80E-05 | ribosomal-protein-serine acetyltransferase |
| SAOUHSC_00376 | -4.6165 | 0.000228 | 0.000246 | hypothetical protein |
| SAOUHSC_00383 | -4.403 | 6.19E-06 | 7.90E-06 | superantigen-like protein |
| SAOUHSC_00398 | -5.2586 | 2.96E-06 | 3.88E-06 | restriction modification system specificity subunit |
| SAOUHSC_00400 | -4.7816 | 8.71E-05 | 9.92E-05 | hypothetical protein |
| SAOUHSC_00401 | -4.2889 | 0 | 0 | hypothetical protein |
| SAOUHSC_00404 | -4.5721 | 0.000291 | 0.000309 | hypothetical protein |
| SAOUHSC_00405 | -3.5928 | 2.36E-27 | 8.07E-27 | hypothetical protein |
| SAOUHSC_00406 | -5.6246 | 7.29E-73 | 5.17E-72 | hypothetical protein |
| SAOUHSC_00408 | -1.7576 | 7.05E-06 | 8.96E-06 | hypothetical protein |
| SAOUHSC_00437 | -1.1265 | 1.51E-08 | 2.42E-08 | hypothetical protein |
| SAOUHSC_00438 | -1.2782 | 5.40E-05 | 6.35E-05 | alpha amylase family protein |
| SAOUHSC_00459 | -1.2734 | 0.000119 | 0.000134 | hypothetical protein |
| SAOUHSC_00499 | -1.9915 | 8.11E-259 | 2.23E-257 | pyridoxal biosynthesis lyase PdxS |
| SAOUHSC_00500 | -1.9787 | 1.28E-201 | 2.90E-200 | glutamine amidotransferase subunit PdxT |
| SAOUHSC_00520 | -1.4255 | 1.13E-56 | 6.80E-56 | 50S ribosomal protein L10 |
| SAOUHSC_00521 | -1.4215 | 9.98E-69 | 6.81E-68 | 50S ribosomal protein L7/L12 |
| SAOUHSC_00529 | -1.0014 | 0 | 0 | elongation factor G |
| SAOUHSC_00585 | -3.9984 | 0.003941 | 0.003519 | hypothetical protein |
| SAOUHSC_00586 | -5.405 | 8.51E-07 | 1.18E-06 | hypothetical protein |
| SAOUHSC_00587 | -5.015 | 1.88E-05 | 2.32E-05 | hypothetical protein |
| SAOUHSC_00639 | -2.6728 | 4.03E-24 | 1.27E-23 | hypothetical protein |
| SAOUHSC_00655 | -1.0047 | 5.97E-17 | 1.46E-16 | dihydroxyacetone kinase subunit DhaK |
| SAOUHSC_00660 | -1.3915 | 1.95E-14 | 4.24E-14 | hypothetical protein |
| SAOUHSC_00697 | -1.9726 | 0.002523 | 0.00234 | hypothetical protein |
| SAOUHSC_00703 | -1.4623 | 2.61E-28 | 9.21E-28 | quinolone resistance protein NorA |
| SAOUHSC_00705 | -1.479 | 0.000424 | 0.000442 | hypothetical protein |
| SAOUHSC_00717 | -1.2962 | 8.64E-35 | 3.50E-34 | hypothetical protein |
| SAOUHSC_00738 | -1.2623 | 2.75E-48 | 1.47E-47 | hypothetical protein |
| SAOUHSC_00818 | -1.6289 | 2.31E-11 | 4.21E-11 | thermonuclease |
| SAOUHSC_00830 | -1.2015 | 3.62E-17 | 8.95E-17 | hypothetical protein |
| SAOUHSC_00864 | -2.3298 | 5.41E-05 | 6.35E-05 | hypothetical protein |
| SAOUHSC_00866 | -1.2095 | 5.73E-19 | 1.52E-18 | D-isomer specific 2-hydroxyacid dehydrogenase NAD binding domain-containing protein |
| SAOUHSC_00871 | -1.3953 | 0.002718 | 0.002511 | D-alanine--poly(phosphoribitol) ligase subunit 2 |
| SAOUHSC_00898 | -2.9648 | 0 | 0 | argininosuccinate lyase |
| SAOUHSC_00899 | -2.2717 | 0 | 0 | argininosuccinate synthase |
| SAOUHSC_00901 | -1.6248 | 1.10E-21 | 3.25E-21 | hypothetical protein |
| SAOUHSC_00902 | -2.0156 | 2.20E-14 | 4.77E-14 | signal peptidase IA |
| SAOUHSC_00903 | -1.1183 | 3.30E-10 | 5.78E-10 | signal peptidase IB |
| SAOUHSC_00924 | -1.5314 | 1.20E-10 | 2.15E-10 | hypothetical protein |
| SAOUHSC_00925 | -1.2102 | 0.000254 | 0.000273 | hypothetical protein |
| SAOUHSC_00972 | -4.742 | 0.000111 | 0.000125 | hypothetical protein |
| SAOUHSC_01005 | -1.328 | 1.22E-135 | 1.63E-134 | hypothetical protein |
| SAOUHSC_01021 | -1.1393 | 2.23E-17 | 5.60E-17 | hypothetical protein |
| SAOUHSC_01022 | -1.2207 | 1.59E-11 | 2.94E-11 | hypothetical protein |
| SAOUHSC_01053 | -2.8458 | 4.41E-107 | 4.49E-106 | manganese transport protein MntH |
| SAOUHSC_01113 | -2.4752 | 3.19E-14 | 6.79E-14 | hypothetical protein |
| SAOUHSC_01114 | -1.2075 | 2.19E-05 | 2.68E-05 | fibrinogen-binding protein |
| SAOUHSC_01121 | -4.1632 | 0 | 0 | alpha-hemolysin |
| SAOUHSC_01115 | -1.8878 | 8.35E-05 | 9.54E-05 | hypothetical protein |
| SAOUHSC_01128 | -1.72 | 6.84E-74 | 4.89E-73 | ornithine carbamoyltransferase |
| SAOUHSC_01129 | -1.8347 | 1.28E-245 | 3.42E-244 | carbamate kinase |
| SAOUHSC_01142 | -2.4371 | 1.58E-93 | 1.47E-92 | cell division protein MraZ |
| SAOUHSC_01130 | -1.6994 | 1.70E-212 | 4.05E-211 | hypothetical protein |
| SAOUHSC_01180 | -1.3272 | 6.55E-17 | 1.59E-16 | hypothetical protein |
| SAOUHSC_01209 | -1.1529 | 0.000161 | 0.000177 | 16S rRNA-processing protein RimM |
| SAOUHSC_01210 | -1.4215 | 4.60E-08 | 7.08E-08 | tRNA (guanine-N(1)-)-methyltransferase |
| SAOUHSC_01214 | -1.5245 | 5.39E-13 | 1.06E-12 | ribosomal biogenesis GTPase |
| SAOUHSC_01215 | -1.3683 | 7.08E-07 | 9.82E-07 | ribonuclease HII |
| SAOUHSC_01266 | -1.0713 | 6.45E-21 | 1.85E-20 | hypothetical protein |
| SAOUHSC_01276 | -1.1198 | 4.82E-21 | 1.39E-20 | glycerol kinase |
| SAOUHSC_01278 | -1.0619 | 7.99E-29 | 2.84E-28 | aerobic glycerol-3-phosphate dehydrogenase |
| SAOUHSC_01284 | -1.3128 | 3.33E-07 | 4.76E-07 | hypothetical protein |
| SAOUHSC_01287 | -1.2442 | 0 | 0 | glutamine synthetase |
| SAOUHSC_01305 | -4.2445 | 0.00144 | 0.0014 | hypothetical protein |
| SAOUHSC_01316 | -1.8372 | 0.000219 | 0.000237 | thermonuclease |
| SAOUHSC_01330 | -1.977 | 3.47E-08 | 5.40E-08 | guanosine 5'-monophosphate oxidoreductase |
| SAOUHSC_01392 | -1.2399 | 6.98E-33 | 2.72E-32 | ABC transporter ATP-binding protein |
| SAOUHSC_01396 | -1.1736 | 0.00012 | 0.000134 | dihydrodipicolinate synthase |
| SAOUHSC_01448 | -1.752 | 2.30E-17 | 5.76E-17 | hypothetical protein |
| SAOUHSC_01450 | -1.9541 | 1.02E-43 | 5.04E-43 | hypothetical protein |
| SAOUHSC_01451 | -3.2935 | 3.68E-137 | 5.14E-136 | threonine dehydratase |
| SAOUHSC_01452 | -3.015 | 8.88E-38 | 3.85E-37 | alanine dehydrogenase |
| SAOUHSC_01482 | -1.2383 | 1.95E-05 | 2.40E-05 | 3-dehydroquinate synthase |
| SAOUHSC_01507 | -1.8719 | 6.35E-05 | 7.38E-05 | hypothetical protein |
| SAOUHSC_01508 | -6.8533 | 1.09E-07 | 1.62E-07 | hypothetical protein |
| SAOUHSC_01524 | -6.4203 | 2.86E-11 | 5.14E-11 | holin-like protein |
| SAOUHSC_01601 | -3.0898 | 3.68E-177 | 7.27E-176 | alpha-D-1%2C4-glucosidase |
| SAOUHSC_01602 | -7.1679 | 0 | 0 | maltose operon transcriptional repressor |
| SAOUHSC_01604 | -1.6882 | 1.93E-17 | 4.88E-17 | hypothetical protein |
| SAOUHSC_01658 | -1.2328 | 1.72E-15 | 3.90E-15 | endonuclease IV |
| SAOUHSC_01659 | -1.155 | 2.86E-26 | 9.55E-26 | hypothetical protein |
| SAOUHSC_01688 | -1.6452 | 1.66E-52 | 9.37E-52 | GTP-binding protein LepA |
| SAOUHSC_01738 | -1.2631 | 3.59E-07 | 5.09E-07 | histidyl-tRNA synthetase |
| SAOUHSC_01749 | -1.0611 | 0.000626 | 0.000636 | S-adenosylmethionine:tRNA ribosyltransferase-isomerase |
| SAOUHSC_01750 | -1.0028 | 2.25E-07 | 3.26E-07 | Holliday junction DNA helicase RuvB |
| SAOUHSC_01751 | -1.2999 | 7.09E-08 | 1.07E-07 | Holliday junction DNA helicase RuvA |
| SAOUHSC_01755 | -1.1141 | 3.26E-37 | 1.40E-36 | 50S ribosomal protein L27 |
| SAOUHSC_01757 | -1.8675 | 1.98E-136 | 2.73E-135 | 50S ribosomal protein L21 |
| SAOUHSC_01761 | -2.569 | 4.79E-112 | 5.17E-111 | hypothetical protein |
| SAOUHSC_01761a | -3.8109 | 0.000386 | 0.000405 | membrane protein |
| SAOUHSC_01824 | -1.2612 | 0.000286 | 0.000305 | thiamine biosynthesis protein ThiI |
| SAOUHSC_01836 | -1.3848 | 4.67E-17 | 1.15E-16 | hypothetical protein |
| SAOUHSC_01875 | -1.3814 | 7.97E-28 | 2.78E-27 | leucyl-tRNA synthetase |
| SAOUHSC_01876 | -1.3109 | 5.79E-20 | 1.60E-19 | hypothetical protein |
| SAOUHSC_01939 | -3.7964 | 2.73E-08 | 4.78E-08 | serine protease SplC |
| SAOUHSC_01919 | -1.1788 | 4.09E-41 | 1.87E-40 | hypothetical protein |
| SAOUHSC_01941 | -3.2598 | 3.60E-04 | 4.22E-04 | serine protease SplB |
| SAOUHSC_01942 | -4.4034 | 1.33E-04 | 1.66E-04 | serine protease SplA |
| SAOUHSC_01957 | -2.9704 | 2.65E-42 | 1.26E-41 | hypothetical protein |
| SAOUHSC_01972 | -1.7432 | 9.26E-242 | 2.40E-240 | protein export protein PrsA |
| SAOUHSC_01990 | -2.169 | 1.37E-46 | 7.11E-46 | amino acid ABC transporter ATP-binding protein |
| SAOUHSC_01991 | -1.3241 | 1.73E-39 | 7.67E-39 | ABC transporter permease |
| SAOUHSC_01992 | -1.0796 | 3.23E-13 | 6.47E-13 | PTS system transporter subunit IIC domain-containing protein |
| SAOUHSC_02012 | -1.8975 | 7.88E-145 | 1.15E-143 | glycosyltransferase |
| SAOUHSC_02014 | -1.6923 | 5.99E-05 | 6.99E-05 | hypothetical protein |
| SAOUHSC_02015 | -5.0315 | 1.67E-05 | 2.08E-05 | hypothetical protein |
| SAOUHSC_02019 | -8.813 | 4.91E-42 | 2.57E-41 | autolysin |
| SAOUHSC_02033 | -4.9297 | 3.37E-05 | 4.08E-05 | phage tape measure protein |
| SAOUHSC_02085 | -5.1868 | 5.25E-06 | 6.74E-06 | hypothetical protein |
| SAOUHSC_02089 | -4.7218 | 0.000125 | 0.000139 | phage family integrase |
| SAOUHSC_02099 | -1.2527 | 7.96E-17 | 1.93E-16 | histidine kinase |
| SAOUHSC_02100 | -1.5743 | 1.24E-11 | 2.29E-11 | hypothetical protein |
| SAOUHSC_02101 | -2.2962 | 3.42E-15 | 7.70E-15 | hypothetical protein |
| SAOUHSC_02112 | -2.199 | 1.82E-40 | 8.17E-40 | hypothetical protein |
| SAOUHSC_02113 | -1.9796 | 5.83E-35 | 2.37E-34 | RNA methyltransferase |
| SAOUHSC_02119 | -1.7661 | 7.83E-50 | 4.30E-49 | high affinity proline permease |
| SAOUHSC_02248 | -6.6596 | 6.45E-14 | 1.34E-13 | hypothetical protein |
| SAOUHSC_02249 | -5.9384 | 3.26E-09 | 5.49E-09 | hypothetical protein |
| SAOUHSC_02250 | -1.8379 | 2.10E-05 | 2.58E-05 | phage terminase small subunit |
| SAOUHSC_02261 | -2.2777 | 2.80E-35 | 1.63E-34 | accessory gene regulator protein B |
| SAOUHSC_02264 | -5.9723 | 0 | 0 | accessory gene regulator protein C |
| SAOUHSC_02265 | -6.3987 | 1.39E-56 | 1.35E-55 | accessory gene regulator protein A |
| SAOUHSC_02281 | -2.5236 | 4.40E-31 | 1.66E-30 | dihydroxy-acid dehydratase |
| SAOUHSC_02282 | -2.472 | 1.40E-08 | 2.26E-08 | acetolactate synthase large subunit |
| SAOUHSC_02284 | -2.7448 | 0.0043 | 0.003813 | ketol-acid reductoisomerase |
| SAOUHSC_02323 | -1.0858 | 2.01E-21 | 5.84E-21 | cardiolipin synthetase |
| SAOUHSC_02324 | -1.968 | 6.89E-15 | 1.53E-14 | hypothetical protein |
| SAOUHSC_02358 | -1.0521 | 2.13E-11 | 3.89E-11 | HemK family modification methylase |
| SAOUHSC_02359 | -1.0879 | 5.71E-16 | 1.32E-15 | peptide chain release factor 1 |
| SAOUHSC_02368 | -1.0924 | 6.96E-16 | 1.60E-15 | CTP synthetase |
| SAOUHSC_02393 | -6.9942 | 7.67E-17 | 1.86E-16 | hypothetical protein |
| SAOUHSC_02396 | -2.2362 | 8.52E-13 | 1.67E-12 | hypothetical protein |
| SAOUHSC_02449 | -2.2212 | 0 | 0 | 6-phospho-beta-galactosidase |
| SAOUHSC_02450 | -2.0654 | 1.30E-230 | 3.27E-229 | PTS system lactose-specific transporter subunit IIBC |
| SAOUHSC_02451 | -1.8069 | 4.84E-15 | 1.08E-14 | PTS system lactose-specific transporter subunit IIA |
| SAOUHSC_02452 | -1.8329 | 1.33E-87 | 1.15E-86 | tagatose 1%2C6-diphosphate aldolase |
| SAOUHSC_02453 | -1.7533 | 2.53E-71 | 1.76E-70 | tagatose-6-phosphate kinase |
| SAOUHSC_02454 | -1.8816 | 4.16E-44 | 2.08E-43 | galactose-6-phosphate isomerase subunit LacB |
| SAOUHSC_02455 | -1.6547 | 5.14E-43 | 2.52E-42 | galactose-6-phosphate isomerase subunit LacA |
| SAOUHSC_02458 | -1.5843 | 0.00109 | 0.001081 | hypothetical protein |
| SAOUHSC_02463 | -2.8703 | 0.000136 | 0.00015 | hyaluronate lyase |
| SAOUHSC_02464 | -6.4239 | 3.66E-12 | 6.95E-12 | hypothetical protein |
| SAOUHSC_02465 | -6.6165 | 1.41E-13 | 2.87E-13 | hypothetical protein |
| SAOUHSC_02471 | -7.6192 | 5.43E-24 | 1.70E-23 | hypothetical protein |
| SAOUHSC_02472 | -8.2232 | 1.24E-33 | 4.92E-33 | hypothetical protein |
| SAOUHSC_02474 | -7.3795 | 5.90E-21 | 1.69E-20 | hypothetical protein |
| SAOUHSC_02475 | -4.4301 | 0.000605 | 0.000619 | hypothetical protein |
| SAOUHSC_02495 | -1.0239 | 3.15E-13 | 6.33E-13 | 50S ribosomal protein L18 |
| SAOUHSC_02496 | -1.0072 | 1.27E-27 | 4.37E-27 | 50S ribosomal protein L6 |
| SAOUHSC_02509 | -1.0201 | 3.70E-101 | 3.65E-100 | 50S ribosomal protein L2 |
| SAOUHSC_02553 | -1.3108 | 0.002347 | 0.0022 | hypothetical protein |
| SAOUHSC_02554 | -1.7783 | 4.49E-11 | 8.12E-11 | hypothetical protein |
| SAOUHSC_02571 | -2.0213 | 4.71E-16 | 1.10E-15 | secretory antigen |
| SAOUHSC_02576 | -1.3136 | 0.00094 | 0.000938 | secretory antigen SsaA |
| SAOUHSC_02579 | -1.9234 | 8.60E-20 | 2.35E-19 | hypothetical protein |
| SAOUHSC_02596 | -1.7913 | 8.14E-22 | 2.41E-21 | hypothetical protein |
| SAOUHSC_02620 | -1.0946 | 4.16E-23 | 1.27E-22 | hypothetical protein |
| SAOUHSC_02622 | -1.2018 | 0.004098 | 0.003641 | sodium/glutamate symporter |
| SAOUHSC_02630 | -1.198 | 0.000408 | 0.000426 | hypothetical protein |
| SAOUHSC_02645 | -1.7303 | 0.003233 | 0.002939 | hypothetical protein |
| SAOUHSC_02661 | -1.8262 | 2.50E-06 | 3.29E-06 | PTS system sucrose-specific transporter subunit IIBC |
| SAOUHSC_02679 | -1.5426 | 7.50E-13 | 1.48E-12 | respiratory nitrate reductase subunit delta |
| SAOUHSC_02680 | -1.5302 | 3.78E-53 | 2.16E-52 | nitrate reductase subunit beta |
| SAOUHSC_02681 | -1.9523 | 1.54E-166 | 2.80E-165 | nitrate reductase subunit alpha |
| SAOUHSC_02682 | -1.223 | 7.95E-44 | 3.96E-43 | uroporphyrin-III C-methyltransferase |
| SAOUHSC_02683 | -1.6588 | 6.02E-09 | 9.85E-09 | assimilatory nitrite reductase [NAD(P)H] small subunit |
| SAOUHSC_02684 | -1.8486 | 4.21E-112 | 4.60E-111 | assimilatory nitrite reductase [NAD(P)H] large subunit |
| SAOUHSC_02685 | -1.8777 | 2.67E-16 | 6.31E-16 | hypothetical protein |
| SAOUHSC_02706 | -2.0555 | 2.26E-10 | 4.59E-10 | immunoglobulin G-binding protein Sbi |
| SAOUHSC_02723 | -1.4362 | 9.37E-37 | 3.97E-36 | glycerate kinase |
| SAOUHSC_02724 | -1.8732 | 8.27E-100 | 8.07E-99 | hypothetical protein |
| SAOUHSC_02729 | -1.0505 | 3.87E-08 | 6.01E-08 | amino acid ABC transporter-like protein |
| SAOUHSC_02756 | -3.2684 | 0.005278 | 0.004613 | hypothetical protein |
| SAOUHSC_02773 | -1.6936 | 4.73E-29 | 1.69E-28 | transporter |
| SAOUHSC_02803 | -2.9247 | 2.25E-06 | 2.98E-06 | fibronectin-binding protein |
| SAOUHSC_02809 | -1.5521 | 0.004616 | 0.004069 | gluconate operon transcriptional repressor |
| SAOUHSC_02815 | -1.9939 | 2.21E-109 | 2.30E-108 | hypothetical protein |
| SAOUHSC_02820 | -3.1754 | 1.52E-10 | 2.70E-10 | hypothetical protein |
| SAOUHSC_02821 | -2.7925 | 1.70E-08 | 2.71E-08 | membrane spanning protein |
| SAOUHSC_02826 | -2.2272 | 2.27E-06 | 3.01E-06 | hypothetical protein |
| SAOUHSC_02829 | -1.6115 | 9.08E-28 | 3.16E-27 | NAD(P)H-flavin oxidoreductase |
| SAOUHSC_02830 | -2.3537 | 1.08E-74 | 7.77E-74 | D-lactate dehydrogenase |
| SAOUHSC_02842 | -2.5901 | 1.11E-30 | 4.12E-30 | hypothetical protein |
| SAOUHSC_02843 | -1.5044 | 5.41E-07 | 7.58E-07 | hypothetical protein |
| SAOUHSC_02848 | -1.1398 | 3.81E-05 | 4.57E-05 | PTS system glucose-specific transporter subunit IIABC |
| SAOUHSC_02856 | -4.1868 | 0.001849 | 0.001767 | hypothetical protein |
| SAOUHSC_02858 | -4.1571 | 0.002096 | 0.001985 | hypothetical protein |
| SAOUHSC_02859 | -1.26 | 3.57E-13 | 7.12E-13 | hydroxymethylglutaryl-CoA reductase |
| SAOUHSC_02872 | -1.8837 | 3.09E-77 | 2.34E-76 | hypothetical protein |
| SAOUHSC_02883 | -2.1329 | 2.17E-33 | 8.56E-33 | LysM domain-containing protein |
| SAOUHSC_02912 | -1.8422 | 8.47E-56 | 5.02E-55 | hypothetical protein |
| SAOUHSC_02913 | -3.9297 | 0.005085 | 0.004461 | hypothetical protein |
| SAOUHSC_02915 | -1.2428 | 0.002432 | 0.002267 | hypothetical protein |
| SAOUHSC_02924 | -1.0628 | 4.15E-05 | 4.94E-05 | 4-aminobutyrate aminotransferase |
| SAOUHSC_02928 | -1.2302 | 0.003231 | 0.002939 | hypothetical protein |
| SAOUHSC_02963 | -2.5384 | 1.45E-17 | 4.14E-17 | clumping factor B |
| SAOUHSC_02964 | -1.3882 | 1.65E-19 | 4.48E-19 | hypothetical protein |
| SAOUHSC_02965 | -1.2092 | 6.61E-14 | 1.37E-13 | carbamate kinase |
| SAOUHSC_02967 | -1.5132 | 2.08E-19 | 5.59E-19 | arginine/ornithine antiporter |
| SAOUHSC_02968 | -1.6438 | 2.93E-12 | 5.59E-12 | ornithine carbamoyltransferase |
| SAOUHSC_02969 | -2.1417 | 4.44E-23 | 1.35E-22 | arginine deiminase |
| SAOUHSC_03025 | -2.5827 | 9.39E-06 | 1.18E-05 | pyrrolidone-carboxylate peptidase |
| SAOUHSC_03041 | -6.6754 | 4.82E-14 | 1.01E-13 | hypothetical protein |
| SAOUHSC_03046 | -2.9445 | 1.79E-06 | 2.39E-06 | helix-turn-helix domain-containing protein |
| SAOUHSC_03051 | -1.2584 | 5.18E-07 | 7.28E-07 | 16S rRNA methyltransferase GidB |
| SAOUHSC_A01081 | -3.756 | 9.40E-07 | 1.30E-06 | hypothetical protein |

**Table S2. Genes differentially expressed (the increased expression) between the VISA and VSSA strains.**

| **Gene id** | **log2.Fold_change.** | **pvalue** | **qvalue** | **Description** |
| --- | --- | --- | --- | --- |
| SAOUHSC_00023 | 1.1267 | 1.22E-10 | 2.19E-10 | hypothetical protein |
| SAOUHSC_00039 | 1.415 | 2.58E-08 | 4.07E-08 | hypothetical protein |
| SAOUHSC_00047 | 2.3199 | 1.21E-07 | 1.81E-07 | hypothetical protein |
| SAOUHSC_00057 | 1.784 | 4.46E-09 | 7.40E-09 | hypothetical protein |
| SAOUHSC_00058 | 2.2091 | 1.50E-13 | 3.06E-13 | hypothetical protein |
| SAOUHSC_00060 | 2.0491 | 1.42E-69 | 9.73E-69 | hypothetical protein |
| SAOUHSC_00065 | 1.7903 | 2.46E-17 | 6.14E-17 | hypothetical protein |
| SAOUHSC_00067 | 1.1073 | 2.78E-14 | 5.97E-14 | L-lactate permease |
| SAOUHSC_00106 | 3.8502 | 1.37E-07 | 2.02E-07 | hypothetical protein |
| SAOUHSC_00114 | 8.0625 | 0 | 0 | capsular polysaccharide biosynthesis protein 5A |
| SAOUHSC_00115 | 7.5579 | 0 | 0 | capsular polysaccharide biosynthesis protein Cap5B |
| SAOUHSC_00116 | 8.2873 | 0 | 0 | capsular polysaccharide biosynthesis protein Cap8C |
| SAOUHSC_00117 | 9.0369 | 0 | 0 | capsular polysaccharide biosynthesis protein Cap5D |
| SAOUHSC_00118 | 8.9985 | 2.08E-88 | 1.89E-87 | capsular polysaccharide biosynthesis protein Cap5E |
| SAOUHSC_00119 | 7.6467 | 0 | 0 | capsular polysaccharide biosynthesis protein Cap8F |
| SAOUHSC_00120 | 1.7473 | 5.26E-37 | 2.25E-36 | UDP-N-acetylglucosamine 2-epimerase |
| SAOUHSC_00125 | 1.7153 | 1.43E-18 | 3.74E-18 | cap5L protein/glycosyltransferase |
| SAOUHSC_00126 | 6.0723 | 7.07E-33 | 2.77E-32 | capsular polysaccharide biosynthesis protein Cap8M |
| SAOUHSC_00127 | 1.2589 | 1.31E-08 | 2.11E-08 | cap5N protein/UDP-glucose 4-epimerase |
| SAOUHSC_00128 | 1.1445 | 3.74E-11 | 6.77E-11 | cap5O protein/UDP-N-acetyl-D-mannosaminuronic acid dehydrogenase |
| SAOUHSC_00129 | 1.1982 | 3.84E-08 | 5.97E-08 | UDP-N-acetylglucosamine 2-epimerase |
| SAOUHSC_00133 | 1.9549 | 1.89E-30 | 6.96E-30 | hypothetical protein |
| SAOUHSC_00135 | 4.8244 | 9.67E-28 | 3.35E-27 | hypothetical protein |
| SAOUHSC_00139 | 2.0626 | 0.00334 | 0.003018 | hypothetical protein |
| SAOUHSC_00143 | 1.2869 | 2.66E-06 | 3.50E-06 | hypothetical protein |
| SAOUHSC_00144 | 1.9173 | 2.13E-277 | 6.2336E-276 | hypothetical protein |
| SAOUHSC_00145 | 1.9917 | 2.36E-63 | 1.51E-62 | hypothetical protein |
| SAOUHSC_00146 | 1.4293 | 1.31E-55 | 7.72E-55 | hypothetical protein |
| SAOUHSC_00152 | 8.8076 | 8.6928E-110 | 9.17E-109 | hypothetical protein |
| SAOUHSC_00164 | 6.9411 | 4.80E-15 | 1.08E-14 | hypothetical protein |
| SAOUHSC_00166 | 6.8552 | 2.56E-14 | 5.54E-14 | hypothetical protein |
| SAOUHSC_00172 | 7.3532 | 5.33E-08 | 8.18E-08 | hypothetical protein |
| SAOUHSC_00173 | 1.6143 | 3.34E-08 | 5.21E-08 | azoreductase |
| SAOUHSC_00229 | 1.0471 | 6.71E-23 | 1.91E-22 | cell wall biosynthesis protein ScdA |
| SAOUHSC_00244 | 2.0783 | 0.003007 | 0.002747 | hypothetical protein |
| SAOUHSC_00255 | 2.5353 | 0.003527 | 0.003181 | hypothetical protein |
| SAOUHSC_00266 | 4.8497 | 0.000163 | 0.000179 | hypothetical protein |
| SAOUHSC_00268 | 6.8988 | 1.11E-14 | 2.44E-14 | hypothetical protein |
| SAOUHSC_00271 | 6.3192 | 1.69E-10 | 3.00E-10 | hypothetical protein |
| SAOUHSC_00285 | 1.4247 | 1.20E-44 | 6.07E-44 | hypothetical protein |
| SAOUHSC_00287 | 1.0616 | 9.71E-20 | 2.65E-19 | ABC transporter ATP-binding protein |
| SAOUHSC_00301 | 2.5244 | 1.31E-06 | 1.77E-06 | hypothetical protein |
| SAOUHSC_00309 | 2.7591 | 9.75E-113 | 1.09E-111 | hypothetical protein |
| SAOUHSC_00322 | 2.0459 | 1.12E-07 | 1.67E-07 | hypothetical protein |
| SAOUHSC_00330 | 5.5891 | 1.62E-07 | 2.36E-07 | hypothetical protein |
| SAOUHSC_00356 | 1.7094 | 3.45E-191 | 1.64E-192 | hypothetical protein |
| SAOUHSC_00367 | 3.4052 | 5.22E-64 | 3.41E-63 | hypothetical protein |
| SAOUHSC_00371 | 1.0231 | 6.87E-122 | 8.09E-121 | hypothetical protein |
| SAOUHSC_00381 | 1.6988 | 0.001995 | 0.001897 | hypothetical protein |
| SAOUHSC_00465 | 1.2521 | 8.62E-48 | 4.60E-47 | hypothetical protein |
| SAOUHSC_00468 | 1.2136 | 1.81E-72 | 1.27E-71 | hypothetical protein |
| SAOUHSC_00469 | 1.3894 | 0 | 0 | regulatory protein SpoVG |
| SAOUHSC_00510 | 1.2081 | 5.36E-05 | 6.31E-05 | serine acetyltransferase |
| SAOUHSC_00511 | 1.1259 | 6.43E-10 | 1.11E-09 | cysteinyl-tRNA synthetase |
| SAOUHSC_00533 | 1.2787 | 5.51E-43 | 2.67E-42 | chaperone protein HchA |
| SAOUHSC_00544 | 2.9992 | 4.01E-29 | 1.44E-28 | fibrinogen-binding protein SdrC |
| SAOUHSC_00545 | 2.1248 | 6.55E-10 | 1.13E-09 | fibrinogen-binding protein SdrD |
| SAOUHSC_00553 | 1.573 | 4.50E-42 | 2.12E-41 | hypothetical protein |
| SAOUHSC_00554 | 1.4114 | 2.86E-29 | 1.03E-28 | SIS domain-containing protein |
| SAOUHSC_00619 | 2.3853 | 2.22E-93 | 2.06E-92 | hypothetical protein |
| SAOUHSC_00620 | 1.139 | 8.93E-157 | 1.50E-155 | accessory regulator A |
| SAOUHSC_00624 | 2.1745 | 3.25E-36 | 1.36E-35 | integrase/recombinase |
| SAOUHSC_00647 | 1.4698 | 5.05E-80 | 3.95E-79 | hypothetical protein |
| SAOUHSC_00674 | 7.3962 | 1.88E-147 | 2.84E-146 | hypothetical protein |
| SAOUHSC_00686 | 1.1877 | 1.60E-22 | 4.82E-22 | hypothetical protein |
| SAOUHSC_00687 | 2.0801 | 1.09E-06 | 1.49E-06 | hypothetical protein |
| SAOUHSC_00689 | 1.6093 | 1.18E-15 | 2.70E-15 | hypothetical protein |
| SAOUHSC_00690 | 1.3533 | 2.20E-96 | 2.10E-95 | hypothetical protein |
| SAOUHSC_00710 | 1.0004 | 0.00216 | 0.002041 | N-acetylglucosamine-6-phosphate deacetylase |
| SAOUHSC_00712 | 1.8874 | 1.15E-87 | 1.01E-86 | hypothetical protein |
| SAOUHSC_00713 | 1.799 | 5.95E-94 | 5.62E-93 | hypothetical protein |
| SAOUHSC_00731 | 1.3195 | 2.66E-21 | 7.68E-21 | ABC transporter |
| SAOUHSC_00733 | 1.2158 | 0.001653 | 0.001587 | histidinol-phosphate aminotransferase |
| SAOUHSC_00736 | 1.1357 | 1.55E-67 | 1.04E-66 | hypothetical protein |
| SAOUHSC_00755 | 1.6868 | 3.08E-25 | 1.00E-24 | hypothetical protein |
| SAOUHSC_00788 | 1.7052 | 5.54E-93 | 5.03E-92 | hypothetical protein |
| SAOUHSC_00789 | 1.6358 | 2.64E-64 | 1.73E-63 | hypothetical protein |
| SAOUHSC_00792 | 1.2568 | 3.66E-34 | 1.47E-33 | hypothetical protein |
| SAOUHSC_00793 | 1.4575 | 6.30E-32 | 2.42E-31 | hypothetical protein |
| SAOUHSC_00802 | 1.1655 | 1.46E-09 | 2.49E-09 | carboxylesterase |
| SAOUHSC_00820 | 1.7662 | 0.003507 | 0.003166 | hypothetical protein |
| SAOUHSC_00824 | 1.3527 | 9.76E-08 | 1.46E-07 | hypothetical protein |
| SAOUHSC_00826 | 1.5685 | 2.22E-268 | 6.31E-267 | hypothetical protein |
| SAOUHSC_00827 | 2.5164 | 3.11E-14 | 6.63E-14 | hypothetical protein |
| SAOUHSC_00831 | 1.2852 | 4.98E-27 | 1.70E-26 | hypothetical protein |
| SAOUHSC_00840 | 2.3887 | 1.71E-25 | 5.62E-25 | hypothetical protein |
| SAOUHSC_00841 | 2.2061 | 2.30E-25 | 7.50E-25 | hypothetical protein |
| SAOUHSC_00845 | 1.5067 | 0 | 0 | hypothetical protein |
| SAOUHSC_00854 | 2.1422 | 1.21E-60 | 7.53E-60 | hypothetical protein |
| SAOUHSC_00855 | 4.1613 | 2.09E-80 | 1.65E-79 | hypothetical protein |
| SAOUHSC_00879 | 4.8684 | 6.02E-24 | 1.88E-23 | cytosol aminopeptidase |
| SAOUHSC_00897 | 1.6781 | 9.41E-13 | 1.83E-12 | hypothetical protein |
| SAOUHSC_00907 | 1.1446 | 7.68E-08 | 1.16E-07 | hypothetical protein |
| SAOUHSC_00936 | 3.1297 | 0.001189 | 0.001171 | hypothetical protein |
| SAOUHSC_00954 | 1.0987 | 5.89E-06 | 7.53E-06 | UDP-N-acetylmuramoylalanyl-D-glutamate--L-lysine ligase |
| SAOUHSC_00961 | 2.0706 | 3.80E-07 | 5.38E-07 | hypothetical protein |
| SAOUHSC_00962 | 3.0035 | 9.48E-76 | 6.99E-75 | hypothetical protein |
| SAOUHSC_00966 | 1.6909 | 1.01E-26 | 3.44E-26 | hypothetical protein |
| SAOUHSC_00986 | 3.852 | 8.70E-48 | 4.62E-47 | cysteine protease |
| SAOUHSC_00987 | 4.1938 | 7.02E-183 | 1.41E-181 | cysteine protease |
| SAOUHSC_00988 | 3.8448 | 3.75E-93 | 3.43E-92 | glutamyl endopeptidase |
| SAOUHSC_01024 | 2.7146 | 0.00054 | 0.000556 | hypothetical protein |
| SAOUHSC_01027 | 1.054 | 1.02E-112 | 1.13E-111 | hypothetical protein |
| SAOUHSC_01049 | 1.373 | 1.92E-21 | 5.58E-21 | spermidine/putrescine ABC transporter substrate-binding protein |
| SAOUHSC_01050 | 1.1969 | 9.34E-63 | 5.93E-62 | hypothetical protein |
| SAOUHSC_01063 | 1.1661 | 2.10E-13 | 4.24E-13 | hypothetical protein |
| SAOUHSC_01091 | 2.2352 | 1.49E-16 | 3.54E-16 | hypothetical protein |
| SAOUHSC_01110 | 2.6238 | 1.01E-23 | 3.58E-23 | fibrinogen-binding protein-like protein |
| SAOUHSC_01135 | 9.9475 | 9.25E-81 | 7.36E-80 | hypothetical protein |
| SAOUHSC_01136 | 6.4712 | 1.83E-11 | 3.35E-11 | hypothetical protein |
| SAOUHSC_01177 | 1.3351 | 9.63E-13 | 1.87E-12 | DNA-directed RNA polymerase subunit omega |
| SAOUHSC_01181 | 1.647 | 6.20E-163 | 1.08E-161 | hypothetical protein |
| SAOUHSC_01248 | 1.6437 | 8.13E-17 | 1.96E-16 | tRNA pseudouridine synthase B |
| SAOUHSC_01264 | 1.2023 | 3.53E-09 | 5.89E-09 | hypothetical protein |
| SAOUHSC_01307 | 2.3677 | 1.85E-05 | 2.29E-05 | hypothetical protein |
| SAOUHSC_01322 | 1.3067 | 4.64E-05 | 5.48E-05 | homoserine kinase |
| SAOUHSC_01326 | 1.7263 | 1.57E-29 | 5.66E-29 | hypothetical protein |
| SAOUHSC_01383 | 1.1619 | 1.79E-08 | 2.86E-08 | hypothetical protein |
| SAOUHSC_01389 | 2.6727 | 5.30E-13 | 1.05E-12 | phosphate ABC transporter substrate-binding protein |
| SAOUHSC_01407 | 1.1616 | 5.28E-07 | 7.41E-07 | hypothetical protein |
| SAOUHSC_01427 | 1.0214 | 1.44E-09 | 2.47E-09 | hypothetical protein |
| SAOUHSC_01435 | 1.341 | 2.86E-41 | 1.32E-40 | thymidylate synthase |
| SAOUHSC_01462 | 1.0462 | 1.25E-23 | 3.87E-23 | hypothetical protein |
| SAOUHSC_01463 | 1.202 | 1.27E-20 | 3.60E-20 | hypothetical protein |
| SAOUHSC_01512 | 4.9556 | 4.52E-09 | 7.45E-09 | hypothetical protein |
| SAOUHSC_01729 | 1.8558 | 4.77E-18 | 1.23E-17 | hypothetical protein |
| SAOUHSC_01730 | 2.1855 | 9.36E-26 | 3.10E-25 | hypothetical protein |
| SAOUHSC_01769 | 1.337 | 2.52E-09 | 4.28E-09 | hypothetical protein |
| SAOUHSC_01787 | 1.304 | 2.00E-25 | 6.54E-25 | hypothetical protein |
| SAOUHSC_01799 | 1.6667 | 2.20E-65 | 1.47E-64 | histidine kinase |
| SAOUHSC_01800 | 1.6443 | 1.24E-27 | 4.29E-27 | alkaline phosphatase synthesis transcriptional regulatory protein |
| SAOUHSC_01815 | 1.3069 | 8.47E-81 | 6.80E-80 | metal-dependent hydrolase |
| SAOUHSC_01855 | 1.4701 | 0 | 0 | hypothetical protein |
| SAOUHSC_01857 | 1.5521 | 1.77E-133 | 2.29E-132 | hypothetical protein |
| SAOUHSC_01858 | 1.4347 | 4.52E-12 | 8.53E-12 | hypothetical protein |
| SAOUHSC_01859 | 1.0423 | 6.87E-07 | 9.56E-07 | hypothetical protein |
| SAOUHSC_01868 | 1.0553 | 2.73E-12 | 5.21E-12 | dipeptidase PepV |
| SAOUHSC_01869 | 1.4191 | 1.17E-87 | 1.02E-86 | hypothetical protein |
| SAOUHSC_01889 | 1.3792 | 0.003797 | 0.0034 | riboflavin biosynthesis protein RibD |
| SAOUHSC_01890 | 2.3024 | 2.04E-17 | 5.16E-17 | hypothetical protein |
| SAOUHSC_01901 | 1.3999 | 2.67E-59 | 1.65E-58 | putative translaldolase |
| SAOUHSC_01902 | 1.8719 | 2.00E-06 | 2.66E-06 | hypothetical protein |
| SAOUHSC_01935 | 1.6319 | 0.001136 | 0.001122 | serine protease SplF |
| SAOUHSC_01936 | 6.6217 | 1.65E-12 | 3.18E-12 | serine protease SplE |
| SAOUHSC_01938 | 3.0917 | 0.005051 | 0.00444 | serine protease SplD |
| SAOUHSC_01947 | 1.9677 | 5.96E-89 | 5.31E-88 | hypothetical protein |
| SAOUHSC_01948 | 2.0203 | 3.82E-128 | 4.69E-127 | ABC transporter |
| SAOUHSC_01949 | 2.8216 | 4.66E-76 | 3.46E-75 | intracellular serine protease |
| SAOUHSC_01950 | 5.0881 | 7.01E-10 | 1.21E-09 | flavoprotein EpiD |
| SAOUHSC_01951 | 5.2256 | 2.14E-32 | 8.31E-32 | epidermin biosynthesis protein EpiC |
| SAOUHSC_01952 | 5.3845 | 1.06E-51 | 5.88E-51 | lantibiotic epidermin biosynthesis protein EpiB |
| SAOUHSC_01958 | 1.4086 | 0.000484 | 0.000502 | hypothetical protein |
| SAOUHSC_01969 | 1.2378 | 1.16E-119 | 1.35E-118 | hypothetical protein |
| SAOUHSC_01971 | 1.653 | 0.000839 | 0.000843 | hypothetical protein |
| SAOUHSC_01986 | 1.5433 | 1.61E-26 | 5.39E-26 | hypothetical protein |
| SAOUHSC_01987 | 1.1229 | 4.82E-129 | 5.98E-128 | hypothetical protein |
| SAOUHSC_02013 | 1.1285 | 3.62E-41 | 1.67E-40 | hypothetical protein |
| SAOUHSC_02095 | 1.0398 | 1.86E-18 | 4.85E-18 | hypothetical protein |
| SAOUHSC_02097 | 1.708 | 8.78E-80 | 6.81E-79 | hypothetical protein |
| SAOUHSC_02138 | 1.5835 | 1.98E-12 | 3.81E-12 | hypothetical protein |
| SAOUHSC_02143 | 1.2067 | 3.31E-09 | 5.55E-09 | hypothetical protein |
| SAOUHSC_02167 | 2.4698 | 1.83E-81 | 1.48E-80 | hypothetical protein |
| SAOUHSC_02181 | 4.6475 | 0.00267 | 0.002469 | phi PVL orfs 18-19-like protein |
| SAOUHSC_02182 | 7.3051 | 3.50E-14 | 7.43E-14 | tail length tape measure protein |
| SAOUHSC_02191 | 5.6853 | 2.84E-07 | 4.08E-07 | HK97 family phage major capsid protein |
| SAOUHSC_02193 | 4.1492 | 0.004502 | 0.003985 | prohead protease |
| SAOUHSC_02200 | 6.1132 | 0.000282 | 0.0003 | hypothetical protein |
| SAOUHSC_02235 | 7.536 | 3.87E-16 | 9.08E-16 | repressor |
| SAOUHSC_02244 | 1.0332 | 3.25E-12 | 6.18E-12 | succinyl-diaminopimelate desuccinylase |
| SAOUHSC_02260 | 13.192 | 0 | 0 | delta-hemolysin |
| SAOUHSC_02262 | 4.9886 | 5.94E-08 | 9.04E-08 | hypothetical protein |
| SAOUHSC_02314 | 1.5627 | 3.80E-06 | 4.95E-06 | sensor protein KdpD |
| SAOUHSC_02315 | 2.5381 | 1.88E-05 | 2.33E-05 | DNA-binding response regulator |
| SAOUHSC_02334 | 1.4702 | 0.000153 | 0.000169 | bacteriophage L54a single-stranded DNA binding protein |
| SAOUHSC_02337 | 1.4295 | 3.88E-52 | 2.17E-51 | UDP-N-acetylglucosamine 1-carboxyvinyltransferase |
| SAOUHSC_02338 | 1.7784 | 1.15E-18 | 3.02E-18 | hypothetical protein |
| SAOUHSC_02363 | 1.3237 | 5.04E-188 | 1.04E-186 | aldehyde dehydrogenase |
| SAOUHSC_02364 | 1.2465 | 1.51E-63 | 9.70E-63 | hypothetical protein |
| SAOUHSC_02381 | 1.0133 | 7.25E-08 | 1.10E-07 | hypothetical protein |
| SAOUHSC_02382 | 1.065 | 1.91E-05 | 2.36E-05 | hypothetical protein |
| SAOUHSC_02387 | 1.5739 | 7.25E-62 | 4.57E-61 | hypothetical protein |
| SAOUHSC_02401 | 1.4632 | 1.66E-23 | 5.12E-23 | hypothetical protein |
| SAOUHSC_02402 | 1.7589 | 1.11E-11 | 2.06E-11 | PTS system mannitol-specific transporter subunit IIA |
| SAOUHSC_02403 | 1.7977 | 1.09E-46 | 5.70E-46 | mannitol-1-phosphate 5-dehydrogenase |
| SAOUHSC_02407 | 1.2459 | 1.22E-19 | 3.30E-19 | hypothetical protein |
| SAOUHSC_02433 | 1.8267 | 1.54E-14 | 3.35E-14 | hypothetical protein |
| SAOUHSC_02434 | 1.7965 | 5.90E-34 | 2.36E-33 | hypothetical protein |
| SAOUHSC_02470 | 2.0174 | 6.85E-05 | 7.94E-05 | hypothetical protein |
| SAOUHSC_02557 | 3.548 | 0.003041 | 0.002776 | urea transporter |
| SAOUHSC_02561 | 3.2496 | 8.44E-11 | 1.52E-10 | urease subunit alpha |
| SAOUHSC_02563 | 1.7577 | 0.00149 | 0.001441 | urease accessory protein UreF |
| SAOUHSC_02565 | 1.7652 | 4.14E-05 | 4.93E-05 | urease accessory protein UreD |
| SAOUHSC_02572 | 1.8522 | 4.66E-61 | 2.92E-60 | hypothetical protein |
| SAOUHSC_02574 | 1.0867 | 2.87E-15 | 6.47E-15 | hypothetical protein |
| SAOUHSC_02589 | 1.6127 | 1.01E-36 | 4.25E-36 | hypothetical protein |
| SAOUHSC_02604 | 1.2056 | 3.48E-53 | 2.01E-52 | hypothetical protein |
| SAOUHSC_02610 | 1.4564 | 3.53E-97 | 3.41E-96 | formimidoylglutamase |
| SAOUHSC_02650 | 1.1787 | 1.01E-198 | 2.17E-197 | hypothetical protein |
| SAOUHSC_02696 | 1.7675 | 1.53E-07 | 2.25E-07 | methicillin resistance determinant protein FmhA |
| SAOUHSC_02700 | 1.038 | 3.38E-05 | 4.08E-05 | hypothetical protein |
| SAOUHSC_02702 | 1.1669 | 0.000964 | 0.000961 | hypothetical protein |
| SAOUHSC_02703 | 1.7401 | 5.03E-43 | 2.48E-42 | phosphoglyceromutase |
| SAOUHSC_02704 | 1.2388 | 3.89E-06 | 5.06E-06 | hypothetical protein |
| SAOUHSC_02751 | 1.9287 | 4.55E-206 | 1.06E-204 | hypothetical protein |
| SAOUHSC_02753 | 1.6307 | 2.76E-30 | 1.01E-29 | hypothetical protein |
| SAOUHSC_02754 | 1.6009 | 2.06E-17 | 5.19E-17 | ABC transporter ATP-binding protein |
| SAOUHSC_02771 | 2.1849 | 3.31E-19 | 8.79E-19 | hypothetical protein |
| SAOUHSC_02772 | 2.3148 | 9.97E-50 | 5.45E-49 | hypothetical protein |
| SAOUHSC_02774 | 2.6797 | 1.63E-163 | 2.90E-162 | hypothetical protein |
| SAOUHSC_02777 | 2.4032 | 5.33E-15 | 1.19E-14 | hypothetical protein |
| SAOUHSC_02779 | 1.8753 | 8.22E-13 | 1.61E-12 | hypothetical protein |
| SAOUHSC_02784 | 4.6853 | 0.000406 | 0.000425 | hypothetical protein |
| SAOUHSC_02802 | 4.2929 | 7.78E-06 | 1.18E-05 | fibronectin binding protein B |
| SAOUHSC_02827 | 2.1885 | 4.84E-54 | 2.81E-53 | hypothetical protein |
| SAOUHSC_02839 | 1.1111 | 2.15E-08 | 3.39E-08 | L-serine dehydratase%2C iron-sulfur-dependent subunit alpha |
| SAOUHSC_02840 | 1.0905 | 0.005016 | 0.004414 | L-serine dehydratase iron-sulfur-dependent subunit beta |
| SAOUHSC_02849 | 1.3691 | 5.59E-152 | 8.74E-151 | pyruvate oxidase |
| SAOUHSC_02850 | 1.5645 | 2.27E-86 | 1.90E-85 | hypothetical protein |
| SAOUHSC_02855 | 1.6605 | 0.00027 | 0.000289 | LysM domain-containing protein |
| SAOUHSC_02862 | 1.087 | 0 | 0 | ATP-dependent Clp protease ATP-binding subunit ClpC |
| SAOUHSC_02877 | 1.8289 | 2.85E-41 | 1.32E-40 | squalene synthase |
| SAOUHSC_02879 | 1.574 | 2.30E-17 | 5.76E-17 | squalene desaturase |
| SAOUHSC_02880 | 2.1215 | 2.11E-76 | 1.58E-75 | hypothetical protein |
| SAOUHSC_02881 | 2.0689 | 1.95E-130 | 2.46E-129 | hypothetical protein |
| SAOUHSC_02882 | 1.5554 | 5.51E-17 | 1.35E-16 | hypothetical protein |
| SAOUHSC_02892 | 2.8098 | 0.000263 | 0.000283 | hypothetical protein |
| SAOUHSC_02899 | 2.2072 | 1.70E-18 | 4.44E-18 | hypothetical protein |
| SAOUHSC_02900 | 1.6073 | 1.28E-38 | 5.62E-38 | hypothetical protein |
| SAOUHSC_02908 | 3.2816 | 3.75E-140 | 5.31E-139 | hypothetical protein |
| SAOUHSC_02925 | 1.2295 | 0.000259 | 0.000278 | hypothetical protein |
| SAOUHSC_02928 | 2.7651 | 5.29E-24 | 1.58E-23 | hypothetical protein |
| SAOUHSC_02926 | 1.6342 | 1.01E-158 | 1.73E-157 | fructose-1%2C6-bisphosphate aldolase |
| SAOUHSC_02929 | 1.3104 | 1.76E-35 | 7.21E-35 | acetyl-CoA synthetase |
| SAOUHSC_02936 | 8.2703 | 4.24E-32 | 1.64E-31 | hypothetical protein |
| SAOUHSC_02941 | 1.7479 | 4.94E-79 | 3.80E-78 | hypothetical protein |
| SAOUHSC_02942 | 2.1852 | 2.07E-300 | 6.71E-299 | anaerobic ribonucleoside triphosphate reductase |
| SAOUHSC_02949 | 2.1454 | 3.48E-06 | 4.54E-06 | hypothetical protein |
| SAOUHSC_02955 | 1.193 | 0.000846 | 0.000849 | nisin susceptibility-associated sensor histidine kinase |
| SAOUHSC_02958 | 2.6784 | 7.04E-05 | 8.14E-05 | alkaline phosphatase III |
| SAOUHSC_02971 | 1.79 | 3.62E-07 | 5.13E-07 | zinc metalloproteinase aureolysin |
| SAOUHSC_02972 | 11.675 | 2.28E-200 | 5.05E-199 | immunodominant antigen B |
| SAOUHSC_02973 | 5.1672 | 2.11E-05 | 2.59E-05 | hypothetical protein |
| SAOUHSC_02975 | 1.3205 | 8.47E-05 | 9.65E-05 | PTS system fructose-specific transporter subunit IIABC |
| SAOUHSC_02983 | 1.4047 | 3.23E-20 | 8.99E-20 | hypothetical protein |
| SAOUHSC_02985 | 1.0456 | 6.20E-17 | 1.51E-16 | preprotein translocase subunit SecA |
| SAOUHSC_02987 | 1.0342 | 0.003304 | 0.002991 | hypothetical protein |
| SAOUHSC_02988 | 1.322 | 0.000864 | 0.000864 | hypothetical protein |
| SAOUHSC_02990 | 2.4194 | 0 | 0 | hypothetical protein |
| SAOUHSC_02991 | 3.8402 | 4.49E-09 | 7.42E-09 | hypothetical protein |
| SAOUHSC_02994 | 1.6927 | 3.84E-56 | 2.29E-55 | hypothetical protein |
| SAOUHSC_02995 | 1.8806 | 4.94E-06 | 6.36E-06 | hypothetical protein |
| SAOUHSC_03001 | 3.0413 | 1.09E-41 | 4.99E-41 | ica operon transcriptional regulator IcaR |
| SAOUHSC_03019 | 3.4702 | 1.75E-06 | 2.35E-06 | ABC transporter ATP-binding protein |
| SAOUHSC_03022 | 1.2608 | 6.15E-47 | 3.22E-46 | hypothetical protein |
| SAOUHSC_03028 | 1.3253 | 3.95E-06 | 5.13E-06 | hypothetical protein |
| SAOUHSC_03032 | 2.3572 | 4.95E-14 | 1.03E-13 | hypothetical protein |
| SAOUHSC_03033 | 1.7094 | 6.65E-08 | 1.01E-07 | high affinity nickel transporter |
| SAOUHSC_03034 | 6.7639 | 1.39E-13 | 2.85E-13 | hypothetical protein |
| SAOUHSC_03035 | 3.9828 | 0 | 0 | hypothetical protein |
| SAOUHSC_03040 | 4.5031 | 0.004583 | 0.004049 | integrase/recombinase |
| SAOUHSC_03045 | 1.0407 | 3.99E-40 | 1.78E-39 | cold shock protein |
